# Diagnostic performance of essential tremor criteria in electronic health records: a retrospective neurology cohort study

**DOI:** 10.3389/fneur.2026.1744336

**Published:** 2026-02-10

**Authors:** Haydn P. Swackhamer, Sonia Stoica, Mikki Sapkota, Oluwaseyi I. Olulana, Mahdi Taye, Akshay C. Patel, Alyssa M. Smith, Iqra C. Mian, Mudit Gupta, Alia C. Stanciu, M. Cosmin Sandulescu

**Affiliations:** 1 Department of Neurology, Geisinger Medical Center, Danville, PA, United States; 2 Department of Neurology, Conemaugh Health System, Johnstown, PA, United States; 3 Department of Neurology, MedStar Health—Georgetown University Hospital, Washington, DC, United States; 4 Geisinger Commonwealth School of Medicine, Scranton, PA, United States; 5 Department of Neurology, Wright State University Boonshoft School of Medicine, Dayton, OH, United States; 6 Healthcare Data Analyst Team Lead, Geisinger Medical Center, Danville, PA, United States; 7 Freeman College of Management, Bucknell University, Lewisburg, PA, United States

**Keywords:** essential tremor, electronic health records, accuracy, sensitivity, specificity, positive predictive value, negative predictive value

## Abstract

**Background:**

The 2018 Movement Disorder Society criteria introduced stricter diagnostic requirements for essential tremor (ET) compared with the 1998 consensus; however, how electronic health record (EHR)–based ET diagnostic coding aligns with chart-adjudicated criteria in routine practice remains unclear. We evaluated the diagnostic performance of EHR-based ET coding relative to chart-adjudicated criteria in a US neurology practice.

**Methods:**

We conducted a retrospective study within the Geisinger Health System in Pennsylvania. Patients with neurologist-assigned ET diagnoses were identified and stratified as ET-only or ET with differential diagnostic coding. A stratified random sample underwent manual chart review. Clinical features were mapped to both criteria after one and two visits. Accuracy metrics—sensitivity, specificity, sample-based positive predictive value (PPV), and sample-based negative predictive value (NPV)—were calculated with 95% confidence intervals.

**Results:**

The reviewed sample included 447 ET-only cases and 137 with differential. With one visit, the 1998 criteria yielded sensitivity of 96% (94–98%), specificity of 60% (53–67%), PPV of 82% (78–86%), and NPV of 88% (82–94%). The 2018 criteria demonstrated similar sensitivity (96%, 94–98%) but lower specificity (38%, 32–44%) and PPV of 53% (48–58%), with NPV of 93% (88–98%). With ≥2 visits, specificity increased to 71% (61–81%) and PPV to 85% (80–90%) for the 1998 criteria, and to 51% (43–59%) and 65% (57–71%) for the 2018 criteria.

**Conclusion:**

In this cohort, ET diagnostic coding showed consistently high sensitivity and NPV across frameworks. Specificity and PPV differed by criterion and improved with multiple clinical encounters.

## 1 Introduction

Essential tremor (ET) is a common diagnosis, with its estimated prevalence ranging from 1.33% in the general population, to 5.9% in adults over 60 (1). ET is a clinical diagnosis, and despite its commonality, ET remains without a clear etiology or diagnostic marker that is pathognomonic for the condition (2). The diagnostic criteria have changed over time and have remained a topic of debate among experts. Essential tremor, as per the 2018 criteria, requires the presentation of bilateral upper extremity action tremor present for at least 3 years, with or without tremors in other locations, and without other neurological signs such as ataxia, dystonia, or parkinsosnim (3). This differs from the 1998 consensus statement that did not put a time constraint on the diagnosis and included isolated head tremor as a possible presentation of ET (4).

This study aims to understand the consistency of ET diagnoses under the new and prior guidelines at one and two visits with a neurologist. Such a comparison can clarify whether changes in diagnostic rules materially affect diagnostic accuracy. If they remain consistent across both criteria, this would support the reliability of neurologist-coded ET diagnoses and validate their use for large-scale epidemiological studies, regardless of the diagnostic era. This is particularly relevant in the Geisinger Health System (GHS), which began adopting electronic health records (EHRs) in the late 1990s and implemented them comprehensively across sites by the early 2000s (5, 6).

Moreover, the absence of comprehensive accuracy metrics on data claim validation, specifically within this population, underscores the importance of this research. One study from 2006 of 71 consecutive patients estimated that 63% of patients with tremors had ET according to 1998 criteria after the specialist visit (7). In a study of 104 consecutive patients from 2020, the term “wastebasket diagnosis” for ET was used to describe the observation that only 45.2% of patients keep this diagnosis made by a primary care or general neurologist before an encounter with a movement disorder specialist using the 2018 diagnostic requirements (8). The most recent study, using International Classification of Diseases (ICD)-10 codes, looked at all the patients over the entire year of 2022 in a tertiary healthcare system, and identified that the positive predictive value (PPV) of ET was 74.7% (9). This higher than previously reported PPV of ET is likely, in part, due to use of the new ICD-10 diagnostic coding over ICD-9 which was less specific in coding for ET (10).

Another key reason for conducting this study is our approach to patient identification, which integrates the International Classification of Diseases (ICD) codes with electronic Differential Diagnoses Generators (DDX) and Epic Diagnosis Groupers (EDG) (11, 12), which gives a unique class for each semantic diagnosis, regardless of the shared ICD. The ICD-9 coding for ET also included drug-induced tremors, enhanced physiologic tremor, psychogenic tremor, and orthostatic tremor in the same 333.1 code, as opposed to the ET-specific ICD-10 code, G25.0 (13). These studies did not solely evaluate neurologists’ accuracy in diagnosing ET but rather evaluated a combination of general neurologists and other specialists who would be expected to use a common diagnosis like ET to describe tremors.

In this study, we evaluated the diagnostic performance of ICD with EDG coded ET diagnoses in a large EHR dataset using structured chart review as a reference standard. We assessed sensitivity, specificity, sample-based positive predictive value, and sample-based negative predictive value of EHR-based ET coding among patients diagnosed by neurologists, including those with and without differential diagnostic coding. A central component of the analysis was comparison of diagnostic performance when chart-documented clinical features were adjudicated using the 1998 and 2018 Movement Disorder Society criteria, applied after one or two neurology visits. By examining how EHR-based ET coding aligns with evolving diagnostic frameworks in routine clinical practice, this work informs the use of large EHR datasets for epidemiologic studies of essential tremor and related movement disorders.

## 2 Methods

This retrospective cohort study was conducted within the Geisinger Health System (GHS), a large integrated healthcare practice in Northeast-Central Pennsylvania, United States. The study period spanned January 2000 to May 2024. We included patients who were ≥18 years old at the time of data extraction. Patients were required to have received an ET diagnosis at age ≥10 years. This threshold was selected to conservatively exclude pediatric tremor presentations more often attributable to metabolic, developmental, or structural causes, while capturing the early-adult-onset peak described in prior epidemiologic studies (14, 15). Eligible patients had at least one ET diagnosis assigned by a neurologist (see Supplementary material 1). Considering the ongoing debate surrounding the suspected link between ET and PD, we decided to exclude patients with an ICD code simultaneously for ET and PD (16, 17) (see Supplementary material 2).

### 2.1 Index test definition (EHR-based diagnostic classification)

The index test consisted of essential tremor (ET)–related diagnostic coding recorded in the electronic health record (EHR). Because a single ET ICD code may coexist with multiple alternative movement disorder diagnoses, we incorporated additional diagnostic context from Epic Diagnosis Groupers (EDG) and Documented Differential Diagnoses (DDX) to refine case classification.

Based on this combined coding framework, two diagnostic classes were constructed. The ICD-ET-only cohort included patients with ET diagnostic codes and no ICD codes corresponding to potential differential diagnoses, including drug-induced tremor, drug-induced Parkinsonism, other tremor syndromes, secondary Parkinsonism, atypical Parkinsonism, physiologic tremor, ataxia, cerebellar ataxia, spinocerebellar atrophy, dystonia, or cervical dystonia (see Supplementary material 3). The ICD-ET with differential diagnosis cohort (ICD-ET+Diff) included patients with ET diagnostic codes and at least one additional ICD code corresponding to a potential differential diagnosis from the same list.

### 2.2 Reference standard (chart-based criteria adjudication)

The reference standard was based on structured manual chart abstraction performed by trained reviewers. Clinical features documented in neurologist notes—including historical characteristics and examination findings—were abstracted using predefined variable definitions. These chart-abstracted data were then mapped algorithmically to diagnostic elements derived from the 1998 and 2018 consensus criteria for essential tremor.

Each diagnostic framework was applied independently to the same chart-abstracted dataset using prespecified rule-based logic, incorporating information from one or two clinical visits as defined in the analysis. Reviewers did not apply diagnostic criteria or assign diagnostic accuracy classes during abstraction and were blinded to ICD-based classification. Diagnostic classifications (true positive, false positive, true negative, and false negative) were generated computationally using R version 4.2.2 (2022-10-31) (18).

### 2.3 Diagnostic performance assessment

Diagnostic performance metrics were calculated in R software using the *epiR* package (19). These metrics reflect the accuracy of EHR-based ET diagnostic coding (index test) relative to chart-based criteria mapping (reference standard), rather than the intrinsic validity of the diagnostic criteria themselves.

To ensure representation of diagnostically complex cases, a stratified random sampling strategy was employed, with patients randomly selected from both the ICD-ET-only and ICD-ET+Diff cohorts. Because this approach did not preserve the prevalence of diagnostic categories in the source population, positive and negative predictive values are reported as sample-based measures and interpreted accordingly.

Abstracted data elements included tremor history (onset, location, type, and symmetry), neurologist-documented examination findings, and whether a differential diagnosis was explicitly recorded in the clinical impression or as an ICD-coded diagnosis. Data were entered into REDCap (Research Electronic Data Capture) (20, 21) using a standardized abstraction form with prespecified definitions for each variable.

Chart abstraction was performed by trained reviewers. Ambiguous or inconsistent documentation was re-reviewed by a movement disorders specialist, with discrepancies resolved by consensus. In addition, a subset of records underwent secondary review by another investigator to ensure consistency of abstraction.

Since this is a retrospective study, we acknowledge that the most we can conclude is a possible diagnosis of essential tremors. For the 1998 criteria patients were considered to have ET if they had (a) reported bilateral action tremor with documentation on neurologist examination, (b) isolated head tremor, or (c) no tremor documented on history, but action tremor documented on exam. When elements on examination were not recorded, they were assumed absent (e.g., if bradykinesia was not mentioned, it was treated as absent). If the nature of tremor onset was undocumented, it was classified as non-sudden (i.e., gradual or intermittent). According to the 2018 criteria, patients were excluded if they had isolated head tremor or bilateral upper-extremity action tremor with a disease duration shorter than three years. We retained individuals with rest tremor or rigidity in the absence of clear bradykinesia in the extremities, recognizing that such features may occur in ET or ET-plus without necessarily indicating Parkinson’s disease. Although ET-plus has been proposed as a diagnostic category, no dedicated ICD-10 code currently exists; therefore, such patients were retained within the ET true-case group.

As a secondary analysis, we restricted the cohort to patients with complete documentation of key examination features (bradykinesia, extremity or gait ataxia, and action tremors) and reclassified the accuracy groups (TP, TN, FP, FN) under this complete-case definition. As a sensitivity analysis for the 2018 criteria, we repeated the calculations excluding cases with undocumented tremor onset date. We then assessed TP and FP rates trajectories by diagnostic criteria and visit numbers and explored chart-based reasons for FP classification.

Descriptive analyses were performed using the Table 1 package in R (22). Continuous variables are summarized as mean and standard deviation (SD) or median and interquartile range (IQR) for non-normal distributions, with between-group comparisons made using two-sample *t*-tests or Wilcoxon rank-sum tests. Categorical variables are reported as counts and percentages, with comparisons made using Chi-squared tests.

**Table 1:** Demographics, comorbidities and data abstraction from chart review by index test classes.

Clinical Data	Level	ICD-ET-only^*^	ICD-ET+Diff ^**^	SMD^***^
Number of patients, n		447	137	
Age of Diagnosis, mean (SD^†^)		58.83 (18.41)	60.02 (17.02)	0.5
Sex, n (%)	Female	237 (53.0)	83 (60.6)	0.1
Race, n (%)	White	435 (97.3)	133 (97.1)	0.7
BMI^‡^, mean (SD^†^)		30.02 (6.74)	29.52 (6.97)	0.4
Hypertension, n (%)	Yes	255 (57.0)	96 (70.1)	< 0.01
Hyperlipidemia, n (%)	Yes	276 (61.7)	84 (61.3)	1
DMT2^§^, n (%)	Yes	116 (26.0)	51 (37.2)	0.01
COPD^∥^, n (%)	Yes	62 (13.9)	36 (26.3)	< 0.01
Asthma, n (%)	Yes	70 (15.7)	28 (20.4)	0.2
Seizures, n (%)	Yes	28 (6.3)	16 (11.7)	0.05
Multiple Sclerosis, n (%)	Yes	8 (1.8)	5 (3.6)	0.3
Ischemic Stroke, n (%)	Yes	11 (2.5)	6 (4.4)	0.3
Tobacco Use, n (%)	Yes	65 (14.5)	26 (19.0)	0.1
	Former	171 (38.5)	47 (34.3)	
	Never	199 (44.5)	64 (46.7)	
	Unknown	11 (2.5)	0 (0.0)	
FHx^††^ of Tremor by ICD, n (%)	Yes	5 (1.1)	4 (2.9)	0.2
FHx^††^ of Parkinsonism by ICD, n (%)	Yes	4 (0.9)	4 (2.9)	0.1
History of Present Illness				
Action Tremor, n (%)	Bilateral	380 (85.0)	123 (89.8)	0.3
	Unilateral	9 (2.0)	1(0.7)	
	Not reported	58 (13)	13 (9.5)	
Years with Tremors, mean (SD)		8.84 (10.58)	10.98 (12.20)	0.04
Onset of Tremor, n (%)	Slowly or intermittent	150 (36.6)	27 (20.3)	< 0.01
	Sudden onset	4 (1.0)	2 (1.5)	
	Not reported	256 (62.4)	104 (78.2)	
Head Tremor, n (%)	Present	134 (30.0)	47 (38.0)	0.6
	Denied	63 (14.1)	18 (13.1)	
	Not documented	250 (55.9)	72 (52.6)	
Memory Complaints, n (%)	Present	142 (31.8)	83 (60.6)	<0.001
	Not present	186 (41.6)	43 (31.4)	
	Not documented	119 (26.6)	11 (8.0)	
Alcohol Sensitivity, n (%)	Yes	42 (9.4)	8 (5.8)	<0.001
	No	33 (7.4)	4 (2.9)	
	Not documented	209 (46.8)	41 (29.9)	
	Denies use	163 (36.5)	84 (61.3)	
FHx^††^ of Tremor, n (%)	Yes	166 (37.1)	40 (29.2)	0.2
	No	101 (22.6)	41(29.9)	
	Not reported	180 (40.3)	56 (40.9)	
FHx^††^ of Parkinsonism, n (%)	Yes	56 (12.5)	21 (15.3)	0.6
	No	50 (11.2)	15 (10.9)	
	Not reported	341 (76.3)	101 (73.7)	
Neurological Examination				
Head Tremor, n (%)	Present	118 (26.4)	46 (33.6)	0.1
	Not present	102 (22.8)	23 (16.8)	
	Not documented	227 (50.8)	68 (49.6)	
Action Tremor, n (%)	Yes	410 (91.7)	134 (97.8)	0.03
	Not present	14 (3.1)	0	
	Not documented	23 (5.1)	3 (2.2)	
Rest Tremor, n (%)	Present	115 (25.7)	71 (51.8)	< 0.001
	Not present	182 (40.7)	37 (27.0)	
	Not documented	150 (33.6)	29 (21.2)	
Bradykinesia, n (%)	Present	14 (3.1)	31 (22.6)	< 0.001
	Not present	184 (41.2)	43 (31.4)	
	Not documented	249 (55.7)	63 (46.0)	
Rigidity, n (%)	Present	38 (8.5)	33 (24.1)	< 0.001
	Not present	284 (63.5)	75 (54.7)	
	Not documented	125 (28.0)	29 (21.2)	
Extremity Ataxia, n (%)	Present	2 (0.4)	29 (21.2)	< 0.001
	Not present	285 (63.8)	63 (46.0)	
	Not documented	160 (35.8)	45 (32.8)	
Abnormal Tandem Gait, n (%)	Present	65 (14.5)	52 (38.0)	< 0.001
	Not present	206 (46.1)	46 (33.6)	
	Not documented	176 (39.4)	39 (28.5)	
Ataxic Gait, n (%)	Present	5 (1.1)	17 (57.7)	< 0.001
	Not present	231 (51.7)	41 (29.9)	
	Not documented	211 (47.2)	79 (57.7)	
Parkinsonian Gait, n (%)	Present	2 (0.4)	2 (1.5)	< 0.001
	Not present	206 (46.1)	30 (21.9)	
	Not documented	239 (53.5)	105 (76.6)	
Impression				
Drug-induced Parkinsonism, n (%)	Yes	5 (1.1)	34 (24.8)	< 0.001
Drug-induced Tremor, n (%)	Yes	18 (4.0)	40 (29.2)	< 0.001
Secondary Tremor, n (%)	Yes	20 (4.5)	48 (35.0)	< 0.001
Parkinson’s Disease, n (%)	Yes	5 (1.1)	20 (14.6)	< 0.001
Atypical Parkinsonism, n (%)	Yes	1 (0.2)	4 (2.9)	0.01

*ICD-ET-only, Essential tremor; ^**^ICD-ET+ICD-Diff, Essential tremor with ICD differential diagnose; ^***^SMD, standardized mean difference; ^†^SD, standard deviation. ^‡^BMI, body mass index; ^§^DMT2, diabetes mellitus type 2; ^∥^COPD, chronic obstructive pulmonary disease; ^††^FHx, family history; ^‡‡^ICD, International Classification of Diseases code; n (%), number patients (percentage).

This study was approved by the Geisinger Medical Center Institutional Review Board (IRB; 2018–0344).

## 3 Results

We identified 7,894 patients with at least one diagnosis of ET by a neurologist. They were ≥18 years old at the time of data extraction and had received an essential tremor (ET) diagnosis at age ≥10 years. We excluded those with an ICD code of PD. The remaining 6,756 patients had ET, of which 826 also had an ICD code for differential diagnosis. 584 patients were randomly selected.

Table 1 presents demographics, comorbidities and data abstraction by essential tremor only codes (ICD-ET-only) and those with codes for ET and differential diagnosis (ICD-ET+Diff).

Compared with ICD-ET-only (*n* = 447), ICD-ET+Diff (*n* = 137) had similar demographics but greater comorbidity burden: hypertension (+13.1 percentage points [pp]; 70.1% vs. 57.0%), type 2 diabetes (+11.2 pp.; 37.2% vs. 26.0%), and COPD (+12.4 pp.; 26.3% vs. 13.9%). ET-typical historical features were less prominent in ICD-ET+Diff (slow/intermittent onset −16.3 pp.; 20.3% vs. 36.6%; alcohol responsiveness −3.6 pp.; 5.8% vs. 9.4%), whereas memory complaints were substantially more common (+28.8 pp.; 60.6% vs. 31.8%). Patients in the ICD-ET+Diff group had a longer tremor duration (10.98 ± 12.20 vs. 8.84 ± 10.58 years). On examination, ICD-ET+Diff demonstrated higher frequencies of parkinsonian signs—rest tremor (+26.1 pp.; 51.8% vs. 25.7%), bradykinesia (+19.5 pp.; 22.6% vs. 3.1%), rigidity (+15.6 pp.; 24.1% vs. 8.5%)—and cerebellar findings—extremity ataxia (+20.8 pp.; 21.2% vs. 0.4%) and abnormal tandem gait (+23.5 pp.; 38.0% vs. 14.5%). Action tremors on exams were nearly universal in both groups (97.8% vs. 91.7%). Clinician impressions more frequently documented non-ET etiologies in the ICD-ET+Diff group, including secondary tremor (+30.5 pp.; 35.0% vs. 4.5%), drug-induced tremor (+25.2 pp.; 29.2% vs. 4.0%), drug-induced parkinsonism (+23.7 pp.; 24.8% vs. 1.1%), and Parkinson’s disease (+13.5 pp.; 14.6% vs. 1.1%). While ICD codes rarely captured family history of tremor or parkinsonism (≤3%), manual chart abstraction identified such documentation in 30–40% of cases.

Compared across 1998 accuracy classes, true positive (TP) and true negative (TN) share similar demographics, and both show high rates of action tremor on exam (97.8 and 97.5%) with predominantly bilateral action tremor reported (91.0 and 90.1%). Differences were observed in comorbidity and neurologic features: TN had higher frequencies of hypertension (66.9% vs. TP 57.6%), COPD (26.4% vs. 14.5%), multiple sclerosis (4.1% vs. 0.8%), and memory complaints (60.3% vs. 32.1%). TN also exhibited higher frequencies of parkinsonian and cerebellar signs on examination, including rest tremor (55.4%), bradykinesia (25.6%), rigidity (24.8%), extremity ataxia (24.0%), abnormal tandem gait (38.8%), and ataxic gait (14.0%), compared with TP (rest tremor 23.6%; other features ≤1%).

Clinician impressions in TN more frequently included drug-induced tremor (33.1%), secondary tremor (39.7%), Parkinson’s disease (16.5%), and drug-induced parkinsonism (28.1%), whereas these impressions were rare in TP. FP differed from TP and TN in showing lower reported bilateral action tremor by history (57%), with a higher proportion of “not reported” entries (39%). Action tremor on examination was less frequent in FP (63.3%), and parkinsonian features were present at intermediate frequencies (rest tremor 35.4%, bradykinesia 17.7%, rigidity 13.9%). Clinician impressions commonly included drug-induced or secondary tremor (22.8 and 24.1%).

FN constituted a small subgroup with higher frequencies of hypertension (93.8%) and diabetes (56.2%), longer symptom duration (14.29 years vs. TN 10.54, TP 9.78, FP 4.43), and frequent memory complaints (62.5%). ET-typical historical features varied across accuracy classes, with TP showing the highest frequency of slow or intermittent onset (38.6%), alcohol responsiveness (11.1%), and documented family history of tremor (40.8% vs. TN 29.8%, FP 20.3%, FN 25.0%). Table 2 summarizes demographics, comorbidities, and chart abstraction findings by 1998 criteria.

**Table 2:** Demographics, comorbidities and data abstraction from chart review by accuracy classes—1998 criteria.

Clinical Data	Level	TP^*^	TN^**^	FP^***^	FN^****^	SMD^§^
Number of patients, n		368	121	79	16	
Age of Diagnosis, mean (SD^†^)		59.02 (18.69)	59.97 (17.34)	57.96 (17.16)	60.44 (14.8)	0.8
Sex, n (%)	Female	194 (52.7)	72 (59.5)	43 (54.4)	11 (68.8)	0.3
Race, n (%)	White	361 (98.1)	117 (96.7)	74 (93.7)	16 (100.0)	0.4
BMI^‡^, mean (SD^†^)		29.98 (6.73)	29.42 (7.04)	30.19 (6.86)	30.25 (6.61)	0.8
Hypertension, n (%)	Yes	212 (57.6)	81 (66.9)	43 (54.4)	15 (93.8)	< 0.01
Hyperlipidemia, n (%)	Yes	230 (62.5)	72 (59.5)	46 (58.2)	12 (75.0)	0.5
DMT2^§§^, n (%)	Yes	98 (26.8)	42 (34.7)	18 (22.8)	9 (56.2)	< 0.05
COPD^∥^, n (%)	Yes	53 (14.5)	32 (26.4)	9 (11.4)	4 (25.0)	< 0.01
Asthma, n (%)	Yes	56 (15.2)	28 (23.1)	14 (17.7)	0 (0.0)	0.06
Seizures, n (%)	Yes	20 (5.4)	15 (12.4)	8 (10.1)	1 (6.2)	0.06
Multiple Sclerosis, n (%)	Yes	3 (0.8)	5 (4.1)	5 (6.3)	0 (0.0)	< 0.01
Ischemic Stroke, n (%)	Yes	10 (2.7)	3 (2.5)	1 (1.3)	3 (18.8)	< 0.01
Tobacco Use, n (%)	Yes	50 (13.6)	21 (17.4)	15 (19.0)	5 (31.2)	< 0.05
	Former	147 (39.9)	43 (35.5)	25 (31.6)	4 (25.0)	
	Unknown	7 (1.9)	0 (0.0)	4 (5.1)	0 (0.0)	
	Never	164 (44.6)	57 (47.1)	35 (44.3)	7 (43.8)	
FHx^††^ of Tremor by ICD, n (%)	Yes	5 (1.4)	3 (2.5)	0 (0.0)	1 (6.2)	0.2
FHx^††^ of Parkinsonism by ICD, n (%)	Yes	3 (0.8)	4 (3.3)	1 (1.3)	0 (0.0)	0.2
History of Present Illness						
Action Tremor, n (%)	Bilateral	335 (91.0)	109 (90.1)	45 (57.0)	14 (87.5)	< 0.001
	Unilateral	6 (1.6)	1 (0.8)	3 (3.8)	0 (0.0)	
	Not reported	27 (7.3)	11 (9.1)	31 (39.2)	2 (12.5)	
Years with Tremors, mean (SD)		9.78 (11.09)	10.54 (10.72)	4.43 (6.10)	14.29 (20.46)	< 0.001
Onset of Tremor, n (%)	Slowly or intermittent	133 (38.6)	23 (19.5)	17 (26.2)	4 (26.7)	<0.001
	Sudden onset	0 (0.0)	2 (1.7)	4 (6.2)	0 (0.0)	
	Not reported	212 (61.4)	93 (78.8)	44 (67.7)	11 (73.3)	
Head Tremor, n (%)	Present	118 (32.1)	44 (36.4)	16 (20.3)	3 (18.8)	0.1
	Not present	54 (14.7)	16 (13.2)	9 (11.4)	2 (12.5)	
	Not documented	195 (53.3)	61 (50.4)	55 (67.9)	11 (68.8)	
Memory Complaints, n (%)	Present	118 (32.1)	73 (60.3)	24 (30.4)	10 (62.5)	<0.001
	Not present	152 (41.3)	40 (33.1)	34 (43.0)	3 (18.8)	
	Not documented	98 (26.6)	8 (6.6)	21 (26.6)	3 (18.8)	
Alcohol Sensitivity, n (%)	Yes	41 (11.1)	7 (5.8)	1 (1.3)	1 (6.2)	<0.001
	No	29 (7.9)	4 (3.3)	4 (5.1)	0 (0.0)	
	Not documented	164 (44.6)	35 (28.9)	45 (57.0)	6 (37.5)	
	Denies use	134 (36.4)	75 (62.0)	29 (36.7)	9 (56.2)	
FHx^††^ of Tremor, n (%)	Yes	150 (40.8)	36 (29.8)	16 (20.3)	4 (25.0)	< 0.01
	No	78 (21.0)	38 (31.4)	23 (29.1)	3 (18.8)	
	Not reported	140 (38.0)	47 (38.8)	40 (50.6)	9 (56.2)	
FHx^††^ of Parkinsonism, n (%)	Yes	47 (12.8)	20 (16.5)	9 (11.4)	1 (6.2)	0.7
	No	40 (10.9)	12 (9.9)	10 (12.7)	3 (18.8)	
	Not reported	281 (76.4)	89 (73.6)	60 (75.9)	12 (75.0)	
Neurological Examination						
Head Tremor, n (%)	Present	105 (28.5)	42 (34.7)	13 (16.5)	4 (25.0)	0.07
	Not present	86 (23.4)	19 (15.7)	16 (20.3)	4 (25.0)	
	Not documented	177 (48.1)	60 (49.6)	50 (63.3)	8 (50.0)	
Action Tremor, n (%)	Yes	360 (97.8)	118 (97.5)	50 (63.3)	16 (100.0)	<0.001
	Not present	4 (1.1)	0 (0.0)	10 (12.7)	0 (0.0)	
	Not documented	4 (1.1)	3 (2.5)	19 (24.1)	0 (0.0)	
Rest Tremor, n (%)	Present	87 (23.6)	67 (55.4)	28 (35.4)	4 (25.0)	<0.001
	Not present	159 (43.2)	29 (24.0)	23 (29.1)	8 (50.0)	
	Not documented	121 (33.2)	25 (20.7)	28 (35.4)	4 (25.0)	
Bradykinesia, n (%)	Present	0 (0.0)	31 (25.6)	14 (17.7)	0 (0.0)	<0.001
	Not present	168 (45.7)	33 (27.3)	16 (20.3)	10 (62.5)	
	Not documented	200 (54.3)	57 (47.1)	49 (62.0)	6 (37.5)	
Rigidity, n (%)	Present	27 (7.3)	30 (24.8)	11 (13.9)	3 (18.8)	<0.001
	Not present	234 (63.6)	65 (53.7)	46 (58.2)	10 (62.5)	
	Not documented	103 (28.0)	26 (21.5)	22 (27.8)	3 (18.8)	
Extremity Ataxia, n (%)	Present	0 (0.0)	29 (24.0)	2 (2.5)	0 (0.0)	<0.001
	Not present	234 (63.6)	52 (43.0)	51 (64.6)	11 (68.8)	
	Not documented	134 (36.4)	40 (33.1)	26 (32.9)	5 (31.2)	
Abnormal Tandem Gait, n (%)	Present	54 (14.7)	47 (38.8)	11 (13.9)	5 (31.2)	<0.001
	Not present	166 (45.1)	39 (32.2)	40 (50.6)	7 (43.8)	
	Not documented	148 (40.2)	35 (28.9)	28 (35.4)	4 (25.0)	
Ataxic Gait, n (%)	Present	0 (0.0)	17 (14.0)	5 (6.3)	0 (0.0)	<0.001
	Not present	197 (53.5)	34 (28.1)	34 (43.0)	7 (43.8)	
	Not documented	171 (46.5)	70 (57.9)	40 (50.6)	9 (56.2)	
Parkinsonian Gait, n (%)	Present	0 (0.0)	2 (1.7)	2 (2.5)	0 (0.0)	<0.001
	Not present	171 (46.5)	25 (20.7)	35 (44.3)	5 (31.2)	
	Not documented	197 (53.5)	94 (77.7)	42 (53.2)	11 (68.8)	
Impression						
Drug-induced Parkinsonism, n (%)	Yes	0 (0.0)	34 (28.1)	5 (6.3)	0 (0.0)	<0.001
Drug-induced Tremor, n (%)	Yes	0 (0.0)	40 (33.1)	18 (22.8)	0 (0.0)	<0.001
Secondary Tremor, n (%)	Yes	1 (0.3)	48 (39.7)	19 (24.1)	0 (0.0)	<0.001
Parkinson’s Disease, n (%)	Yes	0 (0.0)	20 (16.5)	4 (5.1)	0 (0.0)	< 0.001
Atypical Parkinsonism, n (%)	Yes	0 (0.0)	4 (3.3)	1 (1.3)	0 (0.0)	< 0.01

*TP, true positive; ^**^TN, true negative; ^***^FP, false positive; ^****^FN, false negative; ^§^SMD, standardized mean difference; ^†^SD, standard deviation; ^‡^BMI, body mass index; ^§§^DMT2, diabetes mellitus type 2; ^∥^COPD, chronic obstructive pulmonary disease; ^††^FHx, family history; ^‡‡^ICD, International Classification of Diseases code; n (%), number patients (percentage).

Compared across 2018 accuracy classes, TP and TN had similar demographics. Both demonstrated near-universal action tremor on examination (100 and 97.7%), and most reported bilateral action tremor by history (97.0 and 89.8%). TN had higher frequencies of hypertension (68.0% vs. TP 62.1%), diabetes (36.7% vs. 28.9%), COPD (25.8% vs. 15.3%), seizures (11.7% vs. 5.1%), multiple sclerosis (3.9% vs. 0.9%), and memory complaints (60.2% vs. 34.5%). TN also showed higher frequencies of parkinsonian and cerebellar features on examination, including rest tremor (53.1%), bradykinesia (24.2%), rigidity (25.0%), extremity ataxia (22.7%), abnormal tandem gait (37.5%), and ataxic gait (13.3%).

Clinician impressions in TN more frequently included drug-induced tremor (31.2%), secondary tremor (37.5%), drug-induced parkinsonism (26.6%), and Parkinson’s disease (15.6%), whereas these impressions were absent in TP. FP showed lower reported bilateral action tremor by history (71.7% vs. TP 97.0%), a higher proportion of “not reported” entries (24.1%), and reduced action tremor on examination (82.5%). Symptom duration was shortest in FP (2.9 years vs. TP 14.2, TN 10.0, FN 24.5). Parkinsonian signs in FP were present at intermediate frequencies (rest tremor 20.8%, bradykinesia 6.6%, rigidity 7.5%), and clinician impressions included drug-induced or secondary tremor (8.5 and 9.4%).

FN was a small subgroup (*n* = 9) with the most extended tremor duration (24.5 years) and frequent memory complaints (66.7%). On examination, FN demonstrated universal action tremor (100%), occasional rest tremor (33.3%), no bradykinesia, and fewer documented cerebellar or parkinsonian features. ET-typical historical features varied across accuracy classes, with TP showing the highest frequencies of slow or intermittent onset (44.8%), alcohol responsiveness (15.3%), and documented family history of tremor (48.5% vs. TN 28.9%, FP 24.5%, FN 33.3%). Table 3 presents demographics, comorbidities, and chart abstraction findings by 2018 criteria.

**Table 3:** Demographics, comorbidities and data abstraction from chart review by accuracy classes—2018 criteria.

Clinical Data	Level	TP^*^	TN^**^	FP^***^	FN^****^	SMD^§^
Number of patients, n		235	128	212	9	
Age of Diagnosis, mean (SD^†^)		59.88 (17.30)	60.32 (17.14)	57.67 (19.54)	55.78 (15.50)	0.4
Sex, n (%)	Female	123 (52.3)	77 (60.2)	114 (53.8)	6 (66.7)	0.4
Race, n (%)	White	230 (97.9)	124 (96.9)	205 (96.7)	9 (100.0)	0.9
BMI^‡^, mean (SD^†^)		30.39 (6.69)	29.50 (7.05)	29.60 (6.79)	29.78 (6.18)	0.5
Hypertension n, (%)	Yes	146 (62.1)	87 (68.0)	109 (51.4)	9 (100.0)	< 0.01
Hyperlipidemia, n (%)	Yes	150 (63.8)	78 (60.9)	126 (59.4)	6 (66.7)	0.7
DMT2^§§^, n (%)	Yes	68 (28.9)	47 (36.7)	48 (22.6)	4 (44.4)	< 0.05
COPD^∥^, n (%)	Yes	36 (15.3)	33 (25.8)	26 (12.3)	3 (33.3)	< 0.01
Asthma, n (%)	Yes	37 (15.7)	28 (21.9)	33 (15.6)	0 (0.0)	0.2
Seizures, n (%)	Yes	12 (5.1)	15 (11.7)	16 (7.5)	1 (11.1)	0.1
Multiple Sclerosis, n (%)	Yes	2 (0.9)	5 (3.9)	6 (2.8)	0 (0.0)	0.2
Ischemic Stroke, n (%)	Yes	10 (4.3)	4 (3.1)	1 (0.5)	2 (22.2)	< 0.01
Tobacco Use, n (%)	Yes	33 (14.0)	22 (17.2)	32 (15.1)	4 (44.4)	< 0.05
	Former	100 (42.6)	45 (35.2)	72 (34.0)	2 (22.2)	
	Unknown	2 (0.9)	0 (0.0)	9 (4.2)	0 (0.0)	
	Never	100 (42.6)	61 (47.7)	99 (46.7)	3 (33.3)	
FHx^††^ of Tremor by ICD, n (%)	Yes	5 (2.1)	3 (2.3)	0 (0.0)	1 (11.1)	< 0.05
FHx^††^ of Parkinsonism by ICD, n (%)	Yes	3 (1.3)	4 (3.1)	1 (0.5)	0 (0.0)	0.2
History of Present Illness						
Action Tremor, n (%)	Bilateral	228 (97.0)	115 (89.8)	152 (71.7)	8 (88.9)	< 0.001
	Unilateral	0 (0.0)	1 (0.8)	9 (4.2)	0 (0.0)	
	Not reported	7 (3.0)	12 (9.4)	51 (24.1)	1 (11.1)	
Years with Tremors, mean (SD)		14.21 (11.43)	10.03 (10.64)	2.88 (4.90)	24.50 (22.71)	< 0.001
Onset of Tremor, n (%)	Slowly or intermittent	103 (44.8)	24 (19.2)	47 (26.1)	3 (37.5)	< 0.001
	Sudden onset	0 (0.0)	2 (1.6)	4 (2.2)	0 (0.0)	
	Not reported	127 (55.2)	99 (79.2)	129 (71.7)	5 (62.5)	
Head Tremor, n (%)	Present	86 (36.6)	45 (35.2)	48 (22.6)	2 (22.2)	< 0.01
	Not present	40 (17.0)	17 (13.3)	23 (10.8)	1 (11.1)	
	Not documented	109 (46.4)	66 (51.6)	141 (66.5)	6 (66.7)	
Memory Complaints, n (%)	Present	81 (34.5)	77 (60.2)	61 (28.8)	6 (66.7)	< 0.001
	Not present	99 (42.1)	40 (31.2)	87 (41.0)	3 (33.3)	
	Not documented	55 (23.4)	11 (8.6)	64 (30.2)	0 (0.0)	
Alcohol Sensitivity, n (%)	Yes	36 (15.3)	7 (5.5)	6 (2.8)	1 (11.1)	< 0.001
	No	18 (7.7)	4 (3.1)	15 (7.1)	0 (0.0)	
	Not documented	93 (39.6)	38 (29.7)	116 (54.7)	3 (33.3)	
	Denies use	88 (37.4)	79 (61.7)	75 (35.4)	5 (55.6)	
FHx^††^ of Tremor, n (%)	Yes	114 (48.5)	37 (28.9)	52 (24.5)	3 (33.3)	< 0.001
	No	49 (20.9)	39 (30.5)	52 (24.5)	2 (22.2)	
	Not reported	72 (30.6)	52 (40.6)	108 (50.9)	4 (44.4)	
FHx^††^ of Parkinsonism, n (%)	Yes	31 (13.2)	21 (16.4)	25 (11.8)	0 (0.0)	0.6
	No	28 (11.9)	13 (10.2)	22 (10.4)	2 (22.2)	
	Not reported	176 (74.9)	94 (73.4)	165 (77.8)	7 (77.8)	
Neurological Examination						
Head Tremor, n (%)	Present	76 (32.3)	44 (34.4)	42 (19.8)	2 (22.2)	< 0.01
	Not present	62 (26.4)	20 (15.6)	40 (18.9)	3 (33.3)	
	Not documented	97 (41.3)	64 (50.0)	130 (61.3)	4 (44.4)	
Action Tremor, n (%)	Yes	235 (100.0)	125 (97.7)	175 (82.5)	9 (100.0)	< 0.001
	Not present	0 (0.0)	0 (0.0)	14 (6.6)	0 (0.0)	
	Not documented	0 (0.0)	3 (2.3)	23 (10.8)	0 (0.0)	
Rest Tremor, n (%)	Present	71 (30.2)	68 (53.1)	44 (20.8)	3 (33.3)	< 0.001
	Not present	102 (43.4)	31 (24.2)	80 (37.7)	6 (66.7)	
	Not documented	62 (26.4)	29 (22.7)	88 (41.5)	0 (0.0)	
Bradykinesia, n (%)	Present	0 (0.0)	31 (24.2)	14 (6.6)	0 (0.0)	< 0.001
	Not present	116 (49.4)	36 (28.1)	68 (32.1)	7 (77.8)	
	Not documented	119 (50.6)	61 (47.7)	130 (61.3)	2 (22.2)	
Rigidity, n (%)	Present	22 (9.4)	32 (25.0)	16 (7.5)	1 (11.1)	< 0.001
	Not present	158 (67.2)	68 (53.1)	126 (59.4)	7 (77.8)	
	Not documented	55 (23.4)	28 (21.9)	70 (33.)	1 (11.1)	
Extremity Ataxia, n (%)	Present	0 (0.0)	29 (22.7)	2 (0.9)	0 (0.0)	< 0.001
	Not present	154 (65.5)	55 (43.0)	131 (61.8)	8 (88.9)	
	Not documented	81 (34.5)	44 (34.4)	79 (37.3)	1 (11.1)	
Abnormal Tandem Gait, n (%)	Present	37 (15.7)	48 (37.5)	28 (13.2)	4 (44.4)	< 0.001
	Not present	102 (43.4)	43 (33.6)	104 (49.1)	3 (33.3)	
	Not documented	96 (40.9)	37 (28.9)	80 (37.7)	2 (22.2)	
Ataxic Gait, n (%)	Present	0 (0.0)	17 (13.3)	5 (2.4)	0 (0.0)	< 0.001
	Not present	130 (55.3)	36 (28.1)	101 (47.6)	5 (55.6)	
	Not documented	105 (44.7)	75 (58.6)	106 (50.0)	4 (44.4)	
Parkinsonian Gait, n (%)	Present	0 (0.0)	2 (1.6)	2 (0.9)	0 (0.0)	< 0.001
	Not present	112 (47.7)	27 (21.1)	94 (44.3)	3 (33.3)	
	Not documented	123 (52.3)	99 (77.3)	116 (54.7)	6 (66.7)	
Impression						
Drug-induced Parkinsonism, n (%)	Yes	0 (0.0)	34 (26.6)	5 (2.4)	0 (0.0)	< 0.001
Drug-induced Tremor, n (%)	Yes	0 (0.0)	40 (31.2)	18 (8.5)	0 (0.0)	< 0.001
Secondary Tremor, n (%)	Yes	0 (0.0)	48 (37.5)	20 (9.4)	0 (0.0)	< 0.001
Parkinson’s Disease, n (%)	Yes	0 (0.0)	20 (15.6)	5 (2.4)	0 (0.0)	< 0.001
Atypical Parkinsonism, n (%)	Yes	0 (0.0)	4 (3.1)	1 (0.5)	0 (0.0)	< 0.05

*TP, true positive; ^**^TN, true negative; ^***^FP, false positive; ^****^FN, false negative; ^§^SMD, standardized mean difference; ^†^SD, standard deviation; ^‡^BMI, body mass index; ^§§^DMT2, diabetes mellitus type 2; ^∥^COPD, chronic obstructive pulmonary disease; ^††^FHx, family history; ^‡‡^ICD, International Classification of Diseases code; n (%), number patients (percentage).

Table 4 presents sensitivity, specificity, sample-based positive predictive value (PPV), and sample-based negative predictive value (NPV) for essential tremor diagnosis, stratified by at least one versus two or more neurologist visits.

**Table 4:** Essential tremor accuracy results.

Criteria	Sn^†^ (95% CI^*^)	Sp^‡^ (95% CI)	PPV^§^ (95% CI)	NPV^∥^ (95% CI)
At Least One Visit				
1998	96% (94–98%)	60% (53–67%)	82% (78–86%)	88% (82–94%)
2018	96% (93–99%)	38% (32–44%)	53% (48–58%)	93% (88–98%)
2018^**^	96% (93–98%)	39% (34–45%)	54% (50–59%)	93% (88–97%)
At Least Two Visits				
1998	94% (90–98%)	71% (61–81%)	85% (80–90%)	88% (78–94%)
2018	94% (91–97%)	51% (43–59%)	65% (57–71%)	90% (81–96%)

^†^Sn, sensitivity; ^*^CI, confidence interval; ^‡^Sp, specificity; ^§^PPV, sample-based positive predictive value; ^∥^NPV, sample-based negative predictive value; ^**^complete tremor documentation.

Using a conservative approach in which undocumented examination signs were treated as absent, both the 1998 and 2018 criteria demonstrated high sensitivity (approximately 96% at one visit and approximately 94% at two visits) and high sample-based negative predictive values (NPV 88–93%). Sensitivity and NPV were similar across criteria and visit requirements.

Differences between criteria were observed primarily in specificity and sample-based positive predictive value (PPV). At a single visit, the 1998 criteria achieved 60% specificity and 82% PPV, compared with 38% specificity and 53% PPV for the 2018 criteria. Requiring confirmation at a second visit increased specificity and PPV for both criteria, to 71 and 85% for the 1998 criteria and to 51 and 65% for the 2018 criteria, with only modest reductions in sensitivity.

Because this analysis was restricted to patients carrying ET diagnostic codes, individuals without ET codes were not included. Accuracy metrics for the 2018 criteria based on complete tremor onset date (reported onset time) showed slightly higher specificity and PPV.

As a secondary analysis, we restricted the sample to patients (*n* = 141) with complete documentation of bradykinesia, extremity or gait ataxia, and action tremor at least one visit (Table 5). Under this restriction, the 1998 criteria yielded 100 TP, 15 FP, 5 FN, and 21 TN, whereas the 2018 criteria yielded 68 TP, 47 FP, 4 FN, and 22 TN.

**Table 5:** Sensitive analysis for essential tremor with complete records.

Criteria	Sn^†^ (95% CI^*^)	Sp^‡^ (95% CI)	PPV^§^ (95% CI)	NPV^∥^ (95% CI)
1998	95% (89–98%)	58% (41–74%)	87% (79–93%)	81% (61–93%)
2018	94% (86–98%)	32% (21–44%)	59% (50–68%)	85% (65–96%)

^†^Sn, sensitivity; ^*^CI, confidence interval; ^‡^Sp, specificity; ^§^PPV, positive predictive value; ^∥^NPV, negative predictive value.

**Figure 1:**
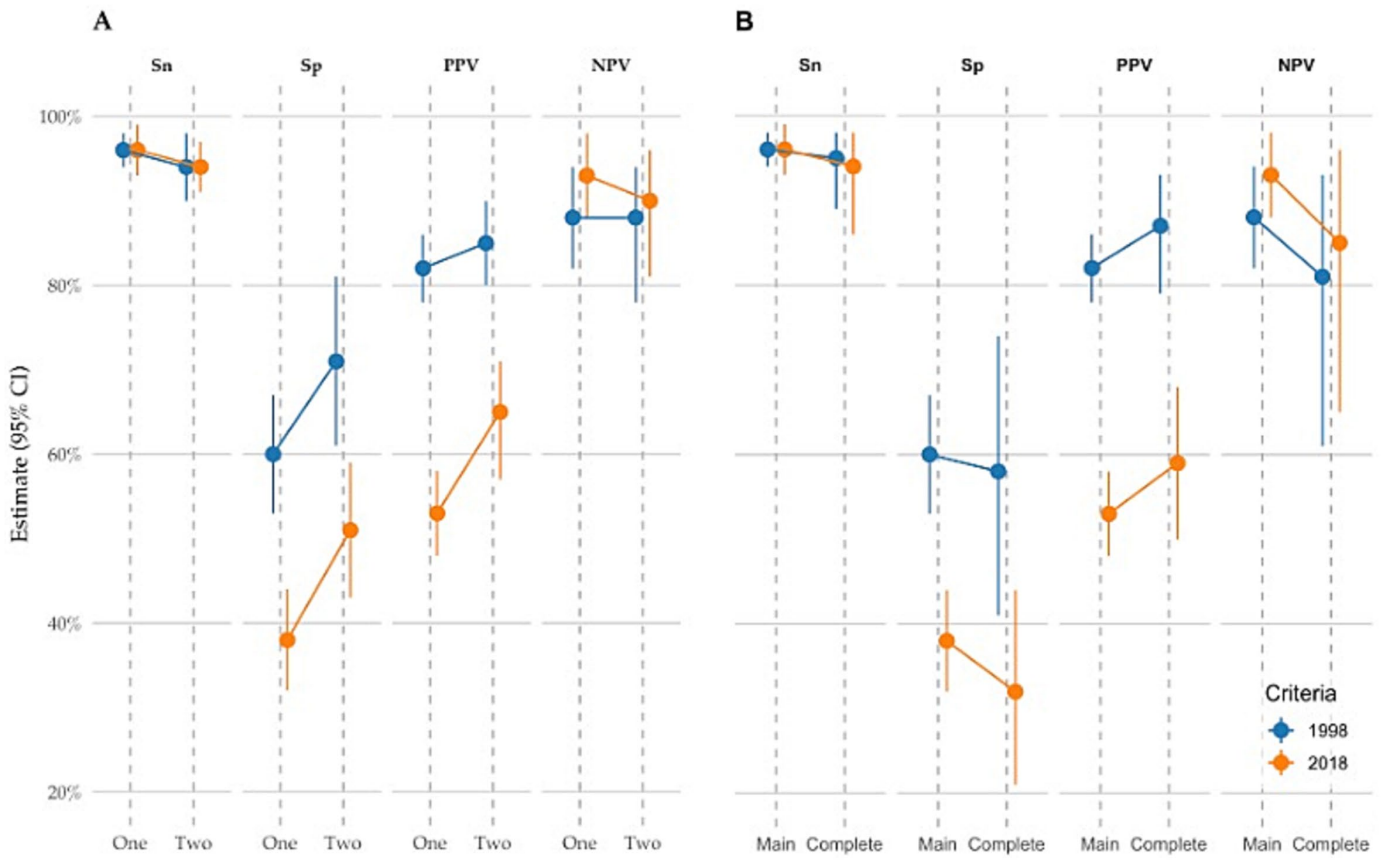
Graphic representation of diagnostic accuracy estimates. **(A)** Results by one versus at least two visits. **(B)** Results from the main analysis (all charts) and the complete-case analysis (restricted to charts with full documentation). Sn, sensitivity; Sp, specificity; PPV, positive predictive value; NPV, negative predictive value.

Diagnostic performance was similar to the main one-visit analysis (Table 4). Sensitivity remained high for both criteria (1998: 95% vs. 96%; 2018: 94% vs. 96%). Specificity was lower under the complete-case restriction (1998: 58% vs. 60%; 2018: 32% vs. 38%). Sample-based predictive values differed across criteria: PPV increased for the 1998 and 2018 criteria (87% vs. 82, 59% vs. 53%), while NPV was lower in both sets (1998: 81% vs. 88%; 2018: 85% vs. 93%). Figure 1 summarizes accuracy metrics from the main analysis (Table 4) and the complete-case analysis (Table 5).

**Figure 2:**
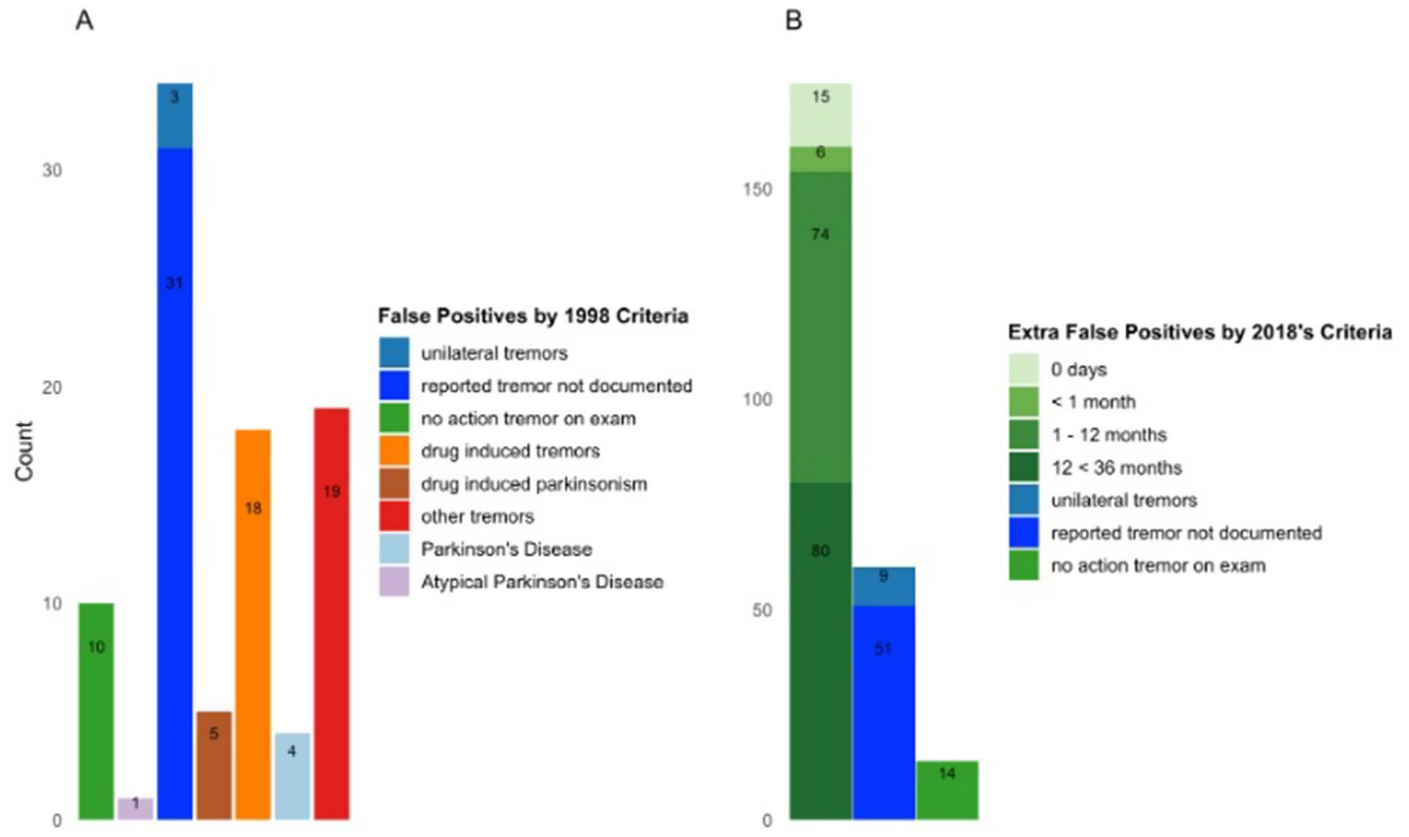
Reasons for false positive cases: **(A)** 1998 criteria; **(B)** 2018 criteria.

Reasons for FP classification differed between the 1998 and 2018 criteria (Figure 2). Under the 1998 criteria (Figure 2A), FP classifications were frequently associated with incomplete documentation, including tremor reported in history but not documented on examination, or with alternative diagnoses such as drug-induced tremor or parkinsonism, other tremor syndromes, or clinical suspicion of Parkinson’s disease or atypical parkinsonism that were not coded as ICD diagnoses. Under the 2018 criteria (Figure 2B), FP classifications more frequently involved absent action tremor, short or uncertain tremor duration, unilateral tremor, or missing documentation. Individual patients could contribute to more than one FP category.

**Figure 3:**
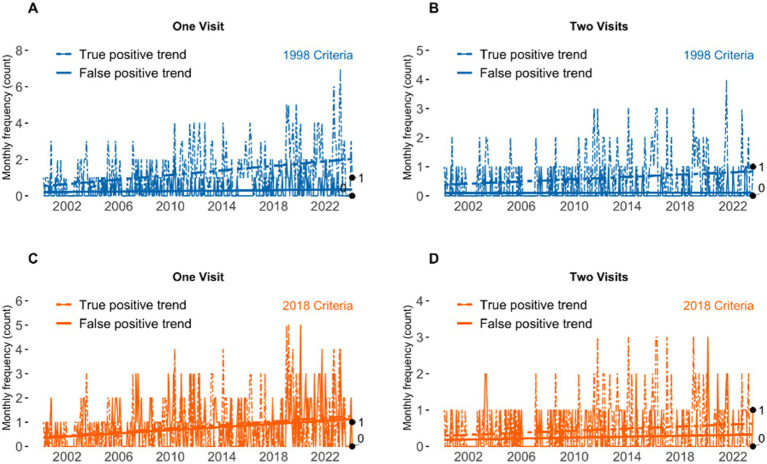
Temporal trends in true positive (TP) and false positive (FP) classifications using the 1998 and 2018 essential tremor diagnostic criteria. Panels show monthly frequencies over calendar time under different diagnostic definitions: **(A)** 1998 criteria requiring one qualifying visit, **(B)** 1998 criteria requiring two qualifying visits, **(C)** 2018 criteria requiring one qualifying visit, and **(D)** 2018 criteria requiring two qualifying visits. Dashed lines represent TP trends and solid lines represent FP trends; fitted trend lines illustrate longitudinal patterns.

Temporal trends differed between the 1998 and 2018 criteria (Figure 3) when at least one visit was considered. Under the 1998 criteria, TP counts increased over time, while FP counts remained stable, both when considering patients with ≥1 visit (Figure 3A) and when restricting to ≥2 visits (Figure 3B). Under the 2018 criteria, both TP and FP counts increased over time when a single visit was required (Figure 3C). When analyses were restricted to patients with ≥2 visits (Figure 3D), FP counts did not increase over time, while TP counts continued to rise modestly. Across all analyses, FP slopes were small in magnitude.

## 4 Discussion

In this large, population-based cohort from a predominantly rural US neurology practice, we evaluated the diagnostic accuracy of EHR–based ET diagnostic coding relative to chart-adjudicated criteria derived from the 1998 and 2018 consensus frameworks, across varying numbers of clinical encounters. Our findings provide several key insights.

High sensitivity and sample-based NPV across criteria. Both the 1998 and 2018 criteria demonstrated very high sensitivity (approximately 94–96%) and negative predictive values (approximately 88–93%) within the chart-reviewed sample, regardless of whether diagnostic accuracy was assessed at ≥1 visit or after ≥2 visits. Unlike most prior studies that have focused primarily on positive predictive value (7–10), this study additionally reports sensitivity for ET identification using EHR-based diagnostic coding. The combined use of essential tremor ICD and EGD codes, along with differential diagnosis coding patterns, enabled the identification of patients with a high likelihood of ET. Sensitivity and sample-based NPV remained consistently high across sensitivity analyses, including restriction to charts with complete examination documentation and analyses incorporating available onset date, indicating that a single analytic assumption did not drive these findings.

Differences in specificity and sample-based PPV. The primary distinction between diagnostic frameworks was observed in specificity and positive predictive value (PPV). At a single visit, the 1998 criteria achieved 60% specificity and 82% sample-based PPV, whereas the 2018 criteria yielded 38% specificity and 53% PPV. Requiring confirmation at ≥2 visits improved diagnostic precision for both frameworks, increasing specificity to 71% for the 1998 criteria and 51% for the 2018 criteria, and PPV to 85 and 65%, respectively. These differences are consistent with the more stringent requirements of the 2018 criteria, including the requirement for a clearly documented three-year tremor duration and exclusion of isolated head tremor (3). In addition, because the study period spanned 2000–2024, most diagnoses were made under the 1998 framework, before the dissemination of the 2018 criteria. Delayed adoption of updated diagnostic guidelines in routine clinical practice may therefore have contributed to the observed differences. As the analysis was restricted to patients carrying ET diagnostic codes, specificity estimates should be interpreted as reflecting differential diagnostic accuracy within an ET-coded population rather than population-level specificity.

To evaluate the impact of missing documentation on diagnostic performance, we performed a sensitivity analysis restricted to patients with complete documentation of key examination features, including action tremor, bradykinesia, and cerebellar signs. Under this restriction, overall diagnostic performance remained broadly consistent with the main one-visit analysis. Sensitivity remained high for both the 1998 and 2018 criteria, indicating that excluding incompletely documented examinations did not materially affect the identification of true ET cases. Specificity was modestly lower in the complete-case analysis, particularly under the 2018 criteria, reflecting the more stringent exclusionary requirements and the reduced sample size. Sample-based PPV increased for both criteria, whereas NPV declined, consistent with the enrichment of clinically well-documented cases. Taken together, these findings suggest that while incomplete documentation contributes to reduced specificity—especially for the 2018 framework—the central observation of high sensitivity across criteria is robust and not driven solely by missing data.

Profiles of false-positive cases. Our abstraction revealed distinct sources of misclassification. False positives under the 1998 criteria were often attributable to incomplete documentation (undocumented action tremor on exam or bilateral tremor on history) or diagnostic overlap with parkinsonism, drug-induced tremor, or secondary tremors. By contrast, most additional false positives under the 2018 criteria arose from insufficiently documented tremor duration (often <12 months), absence of isolated head tremor as an allowable presentation, or incomplete reporting of action tremor on exam. Notably, in many of these overlap cases, clinicians did recognize alternative etiologies such as drug-induced tremor or secondary parkinsonism in their impressions. Still, these were not consistently assigned ICD codes at discharge, leading to apparent false-positive classification in the coding-based framework.

Trends over time. Longitudinal analyses further clarified these patterns. Under the 1998 criteria, true positives increased significantly over time, while false positives remained stable, regardless of number of visits. Under the 2018 criteria, both true and false positives rose significantly at one visit, with slopes of similar magnitude (TP slope 0.0030 vs. FP slope 0.0027), reflecting early misclassification when duration thresholds are not yet met. The FP slope decreased to near zero, while TPs continued to rise slowly in those with at least two visits. Importantly, when analyses were restricted to patients with ≥2 visits, the TP slopes were highly similar across frameworks (1998: 0.00165, 2018: 0.00118). This suggests that repeated neurological evaluations ensure consistent long-term identification of true ET cases independent of which criteria are applied. Across all analyses, FP slopes remained small in magnitude, underscoring the stabilizing effect of follow-up visits on specificity. Interestingly, patients with an FP diagnosis of ET have a family history of tremors, 20.3% for 1998 and 24.5% for the 2018 criteria. It raises the question of whether the family history contributed to neurologists suspecting ET, given the familial nature of ET (23, 24). The documented family history of tremors in those classified as TP, which stands at 41 or 48.8%, depending on the guidelines, falls within the range of 18–67% reported in prior literature (24–29).

Comparison with prior work. Sample-based PPV estimates under the 1998 criteria in our study (82–85%) were higher than those reported in several prior investigations. For example, Louis et al. reported a PPV of 49% using ICD-9 ET codes, although the extent to which alternative diagnostic codes were available to reclassify false positives was not specified (10). Jain et al. observed a PPV of 63% based on clinical abstraction of 71 consecutive patients (7), and Amlang et al. reported 45% concordance in second-opinion evaluations (8).

Differences across studies likely reflect methodological variation, including cohort definition, availability of differential diagnostic coding, and the clinical context in which diagnoses were assigned. In the present study, ET case identification incorporated both essential tremor ICD codes and differential diagnosis coding patterns, and patients with concurrent Parkinson’s disease diagnostic codes were excluded, which may contribute to higher PPV within the sampled population. In addition, our analysis relied on neurologist-assigned diagnoses documented longitudinally, whereas prior studies varied in diagnostic timing and clinical setting.

Under the 2018 criteria, our sample-based PPV estimates (53–65%) were lower than the 74.7% reported by Howard et al. (9), who examined cases from an academic referral center after dissemination of the updated guidelines. That study focused on diagnoses assigned in 2022—approximately 4 years after publication of the 2018 criteria—and included cases identified through primary care encounters with or without neurology follow-up (9). Differences in practice setting, diagnostic adoption timing, and cohort ascertainment likely account for the observed variation in PPV across studies.

Beyond between-study differences, our findings underscore the importance of incorporating additional diagnostic context when using EHR data to identify essential tremor. Because ET ICD codes may coexist with alternative or evolving movement disorder diagnoses, reliance on ICD codes alone can misclassify cases. By integrating ICD codes with documented differential diagnoses and encounter-level diagnostic context (DDX/EDG), our approach seeks to improve discrimination between likely ET and non-ET presentations at cohort entry. This framework may be helpful for EHR-based epidemiologic studies requiring more precise ET case identification (Supplementary materials 1–3).

Our work reports a lower prevalence of abnormal tandem gait of only 14.7–15.7%, but with the mention that in 40% of cases, we have no documentation in the patient’s chart. One study of 36 subjects reported 50% (30), and a more extensive analysis of 358 patients, 47.2% (31) of abnormal tandem gait. As published previously, 23.6–30.2% of reported rest tremors are close prior ranges, from 18.8 to 29.2% (32, 33). ET is associated with cognitive deficits, particularly in memory and executive function (34, 35). One study reported forgetfulness in 50.4% (34) of patients, and in another study, mild cognitive impairment at baseline was reported in 69.2% of ET patients (36). Our cohort reported 32.1–34.4%, but in 23.4–26.6%, we could not find mentions in the records. Studies have found alcohol sensitivity in 46–67% of ET patients (37, 38). Our work shows a similar positive response to alcohol, 66–68% (41 out of 60 for 1998 criteria and 36 out of 54 of alcohol consumption reports for 2018). We found similar reports of head tremors at 28.7–32.3% as reported in the literature at 26.4–31.9 (33, 39, 40). Our demographic data shows no significant difference between ICD-ET-only and ICD-ET+Diff in age or gender distribution. However, there is a significant difference in terms of comorbidities. Patients with ICD-ET+Diff were more likely to have hypertension, type 2 diabetes mellitus, and COPD.

The study has several limitations. Manual chart abstraction introduces the potential for misclassification, particularly in the setting of incomplete or variable clinical documentation. To mitigate this risk, abstraction was guided by standardized definitions, and ambiguous cases were re-reviewed by a movement disorders specialist; however, formal inter-rater reliability metrics were not calculated, and some abstraction variability may remain. Undocumented examination features were conservatively treated as absent, an assumption that may disproportionately affect specificity estimates.

Because the cohort was derived from a single, predominantly rural health system, generalizability may be limited. In addition, the chart-review cohort did not include patients without an essential tremor diagnosis in the electronic health record; therefore, specificity estimates should be interpreted as reflecting differential diagnostic accuracy among ET-labeled patients rather than population-level specificity. Exclusion of patients with Parkinson’s disease diagnostic codes limits assessment of ET–PD overlap and may overestimate diagnostic accuracy in mixed movement disorder populations; however, this restriction reflects the intended analytic use case of ET cohort identification in EHR-based studies. Finally, the retrospective application of both diagnostic frameworks to the same cohort enabled direct comparison but may attenuate context-specific clinical features associated with the diagnostic era in which each framework was developed.

This study provides a comprehensive evaluation of the diagnostic performance of EHR–based ET coding in a predominantly rural neurology practice, using chart-adjudicated criteria as a reference standard. Across both the 1998 and 2018 consensus frameworks, EHR-based ET coding demonstrated consistently high sensitivity and sample-based negative predictive value. In contrast, specificity and sample-based positive predictive value were lower and improved with confirmation across multiple clinical encounters. These findings highlight the strengths of EHR coding for ET case identification while emphasizing the importance of longitudinal assessment and careful interpretation of specificity and predictive values in EHR-based research.

## Data Availability

The data supporting the findings of this study are available from Geisinger Medical Center; however, restrictions apply to their availability, as the data were used under license for the current research and are not publicly available. Data may be obtained from the corresponding author upon reasonable request and with the permission of Geisinger Medical Center. Requests to access the datasets should be directed to msandulescu1@geisinger.edu.
