# Supplementary material for: Diagnostic performance of essential tremor criteria in electronic health records: a retrospective neurology cohort study
Source: Front Neurol. 2026 Feb 10;17:1744336. doi: 10.3389/fneur.2026.1744336 (PMC12929152; doi:10.3389/fneur.2026.1744336)
Supplement: Supplementary file 1 [file Table_1.docx]

| **Supplementary material 1: ICD^*^, EGD^**^ or DDX^***^ codes for Essential Tremor** | |
| --- | --- |
| **Codes** | **Description** |
| 333.1 | Essential and other specified forms of tremor |
| 3439 | Essential and other specified forms of tremor |
| 30762 | Benign essential tremor |
| 30763 | Essential tremor |
| 30764 | Familial tremor |
| 30765 | Hereditary essential tremor |
| 30766 | Heredofamilial tremor |
| 68895 | Benign essential tremor syndrome |
| 68897 | Tremor, essential |
| 68899 | Tremor, hereditary, benign |
| 104491 | Benign familial tremor |
| 252996 | Disabling essential tremor |
| 534263 | Essential tremor |
| 1353120 | Hereditary essential tremor type 2 |
| 1457539 | Hereditary essential tremor type 1 |
| 1457541 | Hereditary essential tremor type 4 |
| G25.0 | Essential tremor |
| ICD^*^ - International Classification of Diseases, EGD^**^ - Epic Diagnosis Groupers; DDX^***^ - Differential Diagnoses Generators | |
