# Supplementary material for: Diagnostic performance of essential tremor criteria in electronic health records: a retrospective neurology cohort study
Source: Front Neurol. 2026 Feb 10;17:1744336. doi: 10.3389/fneur.2026.1744336 (PMC12929152; doi:10.3389/fneur.2026.1744336)
Supplement: Supplementary file 2 [file Table_2.docx]

| Supplementary material 2: ICD^*^, EGD^**^ or DDX^***^ codes for Parkinson’s Disease | |
| --- | --- |
| Code | **Description** |
| 1101865 | Alzheimer's disease with Parkinson's disease |
| G30.9 | Alzheimer's disease with Parkinson's disease |
| 361344 | Cognitive deficit due to Parkinson's disease |
| 429063 | Mild neurocognitive disorder due to Parkinson's disease |
| 429318 | Probable major neurocognitive disorder due to Parkinson's disease with behavioral disturbance |
| 429319 | Major neurocognitive disorder due to Parkinson's disease, probable, with behavioral disturbance |
| 429320 | Probable major neurocognitive disorder due to Parkinson's disease without behavioral disturbance |
| 429321 | Major neurocognitive disorder due to Parkinson's disease, probable, without behavioral disturbance |
| 429338 | Possible major neurocognitive disorder due to Parkinson's disease |
| 429339 | Major neurocognitive disorder due to Parkinson's disease, possible |
| 485871 | Moderate probable major neurocognitive disorder due to Parkinson's disease with behavioral disturbance |
| 485872 | Severe probable major neurocognitive disorder due to Parkinson's disease with behavioral disturbance |
| 486380 | Mild probable major neurocognitive disorder due to Parkinson's disease without behavioral disturbance |
| 486386 | Mild probable major neurocognitive disorder due to Parkinson's disease with behavioral disturbance (HCC) |
| 486422 | Mild possible major neurocognitive disorder due to Parkinson's disease |
| 486727 | Moderate probable major neurocognitive disorder due to Parkinson's disease without behavioral disturbance |
| 486940 | Severe probable major neurocognitive disorder due to Parkinson's disease without behavioral disturbance |
| 487094 | Moderate possible major neurocognitive disorder due to Parkinson's disease |
| 487215 | Severe possible major neurocognitive disorder due to Parkinson's disease |
| 488766 | Mild major neurocognitive disorder due to Parkinson's disease without behavioral disturbance. |
| 488767 | Major neurocognitive disorder, due to Parkinson's disease, without behavioral disturbance, mild |
| 489043 | Mild major neurocognitive disorder due to Parkinson's disease with behavioral disturbance |
| 489044 | Major neurocognitive disorder, due to Parkinson's disease, with behavioral disturbance, mild |
| 1446939 | Major neurocognitive disorder possibly due to Parkinson's disease |
| 1446940 | Severe major neurocognitive disorder probably due to Parkinson's disease, with behavioral disturbance |
| 1446947 | Severe major neurocognitive disorder probably due to Parkinson's disease, without behavioral disturbance |
| 1446954 | Mild major neurocognitive disorder probably due to Parkinson's disease, with behavioral disturbance |
| 1446955 | Mild major neurocognitive disorder probably due to Parkinson's disease, without behavioral disturbance |
| 1446961 | Major neurocognitive disorder probably due to Parkinson's disease, without behavioral disturbance |
| 1446964 | Moderate major neurocognitive disorder probably due to Parkinson's disease, without behavioral disturbance |
| 1446965 | Major neurocognitive disorder probably due to Parkinson's disease, with behavioral disturbance |
| 1446974 | Major neurocognitive disorder possibly due to Parkinson's disease, without behavioral disturbance |
| 1446979 | Moderate major neurocognitive disorder probably due to Parkinson's disease, with behavioral disturbance |
| 1461526 | Major neurocognitive disorder due to Parkinson's disease without behavioral disturbance |
| 1461528 | Major neurocognitive disorder due to Parkinson's disease with behavioral disturbance |
| 1461531 | Major neurocognitive disorder due to Parkinson's disease |
| 429078 | Mild neurocognitive disorder with Lewy bodies |
| 429304 | Possible major neurocognitive disorder with Lewy bodies |
| 429305 | Major neurocognitive disorder with Lewy bodies, possible |
| 429306 | Probable major neurocognitive disorder with Lewy bodies without behavioral disturbance |
| 429307 | Major neurocognitive disorder with Lewy bodies, probable, without behavioral disturbance |
| 429308 | Probable major neurocognitive disorder with Lewy bodies with behavioral disturbance |
| 429309 | Major neurocognitive disorder with Lewy bodies, probable, with behavioral disturbance |
| 485865 | Mild probable major neurocognitive disorder with Lewy bodies without behavioral disturbance |
| 486445 | Mild probable major neurocognitive disorder with Lewy bodies with behavioral disturbance |
| 486726 | Severe probable major neurocognitive disorder with Lewy bodies without behavioral disturbance |
| 486774 | Severe probable major neurocognitive disorder with Lewy bodies with behavioral disturbance |
| 486904 | Mild possible major neurocognitive disorder with Lewy bodies |
| 486905 | Moderate possible major neurocognitive disorder with Lewy bodies |
| 486906 | Severe possible major neurocognitive disorder with Lewy bodies |
| 486939 | Moderate probable major neurocognitive disorder with Lewy bodies without behavioral disturbance |
| 486979 | Moderate probable major neurocognitive disorder with Lewy bodies with behavioral disturbance |
| 491251 | Mixed Lewy body and subcortical vascular dementia |
| 742.4 | Congenital cerebral ventriculomegaly |
| 493102 | Congenital cerebral ventriculomegaly |
| 487632 | Parkinson's disease with EBS (electrical brain stimulation) |
| 487851 | Parkinson's disease with use of electrical brain stimulation |
| 314176 | Dementia with parkinsonism |
| 314177 | Dementia in Parkinson's disease |
| 314178 | Parkinson's disease dementia |
| 375644 | Dementia due to Parkinson's disease without behavioral disturbance |
| 375651 | Dementia due to Parkinson's disease with behavioral disturbance |
| 1445334 | Dementia associated with Parkinson's disease |
| 1446973 | Major neurocognitive disorder possibly due to Parkinson's disease, with behavioral disturbance |
| 1476264 | Dementia due to Parkinson's disease |
| 1480797 | Dementia due to Parkinson's disease, with mood disturbance |
| 1480806 | Severe dementia due to Parkinson's disease, with anxiety |
| 1480832 | Moderate dementia due to Parkinson's disease |
| 1480839 | Severe dementia due to Parkinson's disease, without behavioral disturbance, psychotic disturbance, mood disturbance, or anxiety |
| 1480876 | Moderate dementia due to Parkinson's disease, with mood disturbance |
| 1480881 | Dementia due to Parkinson's disease, with agitation |
| 1480904 | Dementia due to Parkinson's disease, without behavioral disturbance, psychotic disturbance, mood disturbance, or anxiety |
| 1480912 | Mild dementia due to Parkinson's disease, with mood disturbance |
| 1480927 | Severe dementia due to Parkinson's disease, with mood disturbance |
| 1480932 | Moderate dementia due to Parkinson's disease, with agitation |
| 1480941 | Severe dementia due to Parkinson's disease |
| 1480955 | Dementia due to Parkinson's disease, with psychotic disturbance |
| 1480959 | Dementia due to Parkinson's disease, with anxiety |
| 1480998 | Severe dementia due to Parkinson's disease, with agitation |
| 1481003 | Severe dementia due to Parkinson's disease, with psychotic disturbance |
| 1481021 | Mild dementia due to Parkinson's disease |
| 1481029 | Mild dementia due to Parkinson's disease, with psychotic disturbance |
| 1481038 | Mild dementia due to Parkinson's disease, with anxiety |
| 1481042 | Mild dementia due to Parkinson's disease, with agitation |
| 1481048 | Moderate dementia due to Parkinson's disease, with anxiety |
| 1481053 | Mild dementia due to Parkinson's disease, without behavioral disturbance, psychotic disturbance, mood disturbance, or anxiety |
| 1481091 | Moderate dementia due to Parkinson's disease, without behavioral disturbance, psychotic disturbance, mood disturbance, or anxiety |
| 1481123 | Moderate dementia due to Parkinson's disease, with psychotic disturbance |
| 1482946 | Moderate dementia due to Parkinson's disease, with other behavioral disturbance |
| 1482953 | Severe dementia due to Parkinson's disease, with other behavioral disturbance |
| 1482959 | Mild dementia due to Parkinson's disease, with other behavioral disturbance |
| 1483430 | Mild dementia due to Parkinson's disease, unspecified whether behavioral, psychotic, or mood disturbance or anxiety |
| 1483439 | Moderate dementia due to Parkinson's disease, unspecified whether behavioral, psychotic, or mood disturbance or anxiety |
| 1483451 | Severe dementia due to Parkinson's disease, unspecified whether behavioral, psychotic, or mood disturbance or anxiety |
| 1483475 | Dementia due to Parkinson's disease, with agitation, unspecified dementia severity |
| 1483476 | Dementia due to Parkinson's disease, with anxiety, unspecified dementia severity |
| 1483477 | Dementia due to Parkinson's disease, with mood disturbance, unspecified dementia severity |
| 1483478 | Dementia due to Parkinson's disease, with other behavioral disturbance, unspecified dementia severity |
| 1483479 | Dementia due to Parkinson's disease, without behavioral disturbance, psychotic disturbance, mood disturbance, or anxiety, unspecified dementia severity |
| 1483480 | Dementia due to Parkinson's disease, with psychotic disturbance, unspecified dementia severity |
| 1483520 | Dementia due to Parkinson's disease, unspecified dementia severity, unspecified whether behavioral, psychotic, or mood disturbance or anxiety |
| 532670 | Autosomal recessive early onset Parkinson disease type15 |
| 532671 | Autosomal recessive early onset Parkinson disease associated with mutation in SYNJ1 gene |
| 532672 | Autosomal recessive early onset Parkinson disease type 20 |
| 532673 | Autosomal recessive early onset Parkinson disease associated with mutation in PINK1 gene |
| 532674 | Autosomal recessive early onset Parkinson disease type 6 |
| 532680 | Autosomal recessive juvenile Parkinson disease associated with mutation in PARK2 |
| 532700 | Autosomal recessive juvenile onset Parkinson disease associated with mutation in DNAJC6 gene |
| 532701 | Autosomal recessive juvenile onset Parkinson disease type19 |
| 1274172 | Autosomal dominant late onset Parkinson disease |
| 1477633 | Autosomal recessive familial Parkinson's disease |
| 121252 | Parkinson's disease, Lewy body |
| 128906 | Lewy body Parkinson's disease |
| 128907 | Lewy body Parkinson's disease |
| 324111 | Dyskinesia due to Parkinson's disease |
| 372586 | Levodopa-induced dyskinesia |
| 373612 | Levodopa-induced dyskinesia |
| 67837 | Parkinsonism |
| 1221228 | Parkinsonism, unspecified Parkinsonism type |
| 1350679 | Parkinsonism due to heredodegenerative disorder |
| 528648 | Autosomal dominant Parkinson disease associated with mutation in LRRK2 gene |
| 528649 | Autosomal dominant Parkinson disease type 8 |
| 528650 | Autosomal dominant Parkinson disease associated with mutation in UCHL1 gene |
| 528651 | Autosomal dominant Parkinson disease type 5 |
| 528652 | Autosomal dominant Parkinson disease associated with mutation in EIF4G1 gene |
| 528653 | Autosomal dominant Parkinson disease type 18 |
| 528654 | Autosomal dominant Parkinson disease associated with mutation in VPS35 gene |
| 528655 | Autosomal dominant Parkinson disease type 17 |
| 528656 | Autosomal dominant Parkinson disease associated with mutation in GIGYF2 gene |
| 528657 | Autosomal dominant Parkinson disease type 11 |
| 528658 | Autosomal dominant Parkinson disease associated with mutation in SNCA gene |
| 528659 | Autosomal dominant Parkinson disease type 1 |
| 532336 | Autosomal recessive Parkinson disease associated with mutation in PLA2G6 gene |
| 532337 | Autosomal recessive Parkinson disease type 14 |
| 532338 | Autosomal recessive Parkinson's disease associated with mutation in PARK7 gene |
| 532339 | Autosomal recessive Parkinson's disease type 7 |
| 532681 | Autosomal recessive juvenile Parkinson disease type 2 |
| 1355851 | Autosomal dominant Parkinson's disease type 3 |
| 1355854 | Autosomal recessive juvenile Parkinson's disease |
| 499231 | Motor fluctuations related to medication use in Parkinson's disease |
| 54073 | Parkinson's syndrome |
| 332 | Parkinson disease |
| 3434 | Parkinson's disease |
| 3435 | Parkinson's disease |
| 30735 | Idiopathic Parkinsonism |
| 30736 | Paralysis agitans |
| 30737 | Primary parkinsonism |
| 30738 | Shaking palsy |
| 54072 | Parkinson disease |
| 54074 | Parkinsons disease |
| 54079 | Parkinson disease, symptomatic |
| 54080 | Parkinsonian syndrome |
| 54083 | Symptomatic Parkinson disease |
| 99374 | Idiopathic Parkinson's disease |
| 101301 | PD (Parkinson's disease) |
| 163247 | Juvenile Parkinson's disease |
| 165846 | Parkinsons |
| 201625 | Parkinson's disease (tremor, stiffness, slow motion, unstable posture) |
| 524043 | Nonfamilial idiopathic Parkinson's disease |
| 537017 | Parkinson's disease |
| 1273711 | Young-onset Parkinson's disease |
| 1273737 | Early-onset Parkinson's disease |
| 1348464 | Parkinson's disease not affecting current episode of care |
| 1430982 | Sporadic Parkinson's disease |
| 1433395 | Primary Parkinson's disease |
| 1452404 | Parkinson's disease with levodopa-resistant atypical features |
| 1485663 | Off-periods in Parkinson disease not responding to oral treatment |
| 332.0 | Paralysis agitans |
| G20 | Parkinson's disease |
| 1349086 | Psychosis due to Parkinson's disease |
| ICD^*^ - International Classification of Diseases, EGD^**^ - Epic Diagnosis Groupers; DDX^***^ - Differential Diagnoses Generators | |
