# Supplementary material for: Diagnostic performance of essential tremor criteria in electronic health records: a retrospective neurology cohort study
Source: Front Neurol. 2026 Feb 10;17:1744336. doi: 10.3389/fneur.2026.1744336 (PMC12929152; doi:10.3389/fneur.2026.1744336)
Supplement: Supplementary file 3 [file Table_3.docx]

| Supplementary material 3: ICD^*^, EGD^**^ or DDX^***^ codes for Differential Diagnosis | |
| --- | --- |
| Code | **Description** |
| 331.4 | Acquired cerebral ventriculomegaly |
| 521970 | Acquired cerebral ventriculomegaly |
| 94 | Progressive spinal ataxia |
| 270 | Syndrome, pellagra-cerebellar ataxia-renal aminoaciduria |
| 270.3 | Dilated cardiomyopathy with ataxia syndrome |
| 271.8 | Ataxia with lactic acidosis I |
| 277.89 | Ataxia with lactic acidosis I |
| 285 | Ataxia due to mitochondrial mutations |
| 294.1 | Neurodegenerative disease with dementia, ataxia, and spasticity |
| 330 | Childhood ataxia with diffuse central nervous system hypomyelination syndrome |
| 331.89 | X-linked ataxia, apraxia, and mental retardation |
| 334 | Friedreich's ataxia |
| 334.3 | Hereditary ataxia-muscular atrophy syndrome |
| 334.8 | Ataxia-telangiectasia syndrome |
| 345.1 | POLG-related myoclonic epilepsy, myopathy, and sensory ataxia (MEMSA) |
| 349.89 | Ketoaciduria, intellectual disability, ataxia, and deafness syndrome |
| 356 | Spinocerebellar ataxia type 3 |
| 356.3 | Ataxia due to phytanic acid storage disease |
| 356.8 | Neuropathy in association with hereditary ataxia |
| 356.9 | Neuropathy in association with hereditary ataxia |
| 362.74 | Retinitis pigmentosa-deafness-ataxia syndrome |
| 378.81 | Benign paroxysmal tonic upgaze of childhood with ataxia |
| 379.51 | Ataxia with oculomotor apraxia type 1 |
| 389.9 | Deafness with hyperuricemia and neurologic ataxia |
| 425.8 | Dilated cardiomyopathy secondary to Friedreich's ataxia |
| 753.15 | Renal dysplasia with retinal pigmentary dystrophy, cerebellar ataxia, and skeletal dysplasia |
| 758.9 | Autosomal dominant cerebellar ataxia linked to chromosome 16q |
| 759.83 | Fragile X associated tremor ataxia syndrome |
| 759.89 | Autosomal recessive cerebellar ataxia, pyramidal signs, nystagmus, and oculomotor apraxia syndrome |
| 781.99 | Hypotonia with congenital nystagmus, ataxia, and abnormal brainstem auditory response |
| 3459 | Friedreich's ataxia |
| 3461 | Primary cerebellar degeneration |
| 3462 | Cerebellar ataxia |
| 3464 | Other spinocerebellar diseases |
| 3465 | Spinocerebellar disease |
| 21817 | Dilated cardiomyopathy secondary to Friedreich's ataxia |
| 21843 | Hypertrophic cardiomyopathy secondary to Friedreich's ataxia |
| 22461 | Ataxia-telangiectasia syndrome |
| 30720 | Spinal ataxia |
| 30802 | Familial ataxia |
| 30803 | Friedreich's disease |
| 30804 | Hereditary ataxia |
| 30807 | Hereditary ataxia-muscular atrophy syndrome |
| 30810 | Marie's cerebellar ataxia |
| 30811 | Hereditary cerebellar ataxia |
| 30812 | Sanger-Brown cerebellar ataxia |
| 30813 | Vestibulocerebellar ataxia |
| 30818 | Hereditary cerebellar degeneration |
| 30819 | Hereditary ataxia, unspecified |
| 30822 | Corticostriatal-spinal degeneration |
| 31373 | Retinitis pigmentosa-deafness-ataxia syndrome |
| 31752 | Behr's syndrome I |
| 31753 | Infantile optic atrophy-ataxia |
| 43410 | Ataxia, telangiectasia |
| 43416 | Telangiectasia, ataxic |
| 43417 | Telangiectasia, cerebello-oculocutaneous |
| 44453 | Ataxia, cerebellar |
| 44457 | Cerebellar ataxia/dyskinesia |
| 47838 | Ataxia, familial |
| 47839 | Ataxia, Freidreich's |
| 47840 | Ataxia, Freidriech's |
| 47841 | Ataxia, Friedreich |
| 47842 | Ataxia, Friedreich's |
| 47843 | Ataxia, Friedriech's |
| 47844 | Ataxia, hereditary, spinal |
| 47845 | Ataxia, spinal, hereditary |
| 47846 | Ataxy, familial |
| 47847 | Ataxy, family |
| 47848 | Ataxy, Friedreich's |
| 47849 | Ataxy, hereditary, spinal |
| 47850 | Ataxy, spinal, hereditary |
| 47851 | Freidreich ataxia |
| 47852 | Freidreich's ataxia |
| 47853 | Freidreichs ataxia |
| 47854 | Freidriech ataxia |
| 47855 | Freidriech's ataxia |
| 47856 | Freidriechs ataxia |
| 47857 | Friedreich ataxia |
| 47858 | Friedreich's, ataxia |
| 47859 | Friedreichs ataxia |
| 47860 | Friedriech ataxia |
| 47861 | Friedriech's ataxia |
| 47862 | Friedriechs ataxia |
| 47863 | Hereditary ataxia of Friedreich |
| 47864 | Hereditary spinal ataxia |
| 47865 | Hereditary spinal sclerosis |
| 47866 | Hereditary spinal sclerosis |
| 47867 | Sclerosis, hereditary spinal |
| 47868 | Sclerosis, hereditary spinal |
| 47869 | Sclerosis, hereditary, spinal |
| 47870 | Sclerosis, spinal, hereditary |
| 47871 | Spinal sclerosis, hereditary |
| 47872 | Spinal sclerosis, hereditary |
| 47873 | Ataxia, familial |
| 47874 | Ataxia, Freidreich's |
| 47875 | Ataxia, Freidriech's |
| 47876 | Ataxia, Friedreich |
| 47877 | Ataxia, Friedreich's |
| 47878 | Ataxia, Friedriech's |
| 47879 | Ataxia, hereditary, spinal |
| 47880 | Ataxia, spinal, hereditary |
| 47881 | Ataxy, familial |
| 47882 | Ataxy, family |
| 47884 | Ataxy, hereditary, spinal |
| 47885 | Ataxy, spinal, hereditary |
| 47886 | Freidreich ataxia |
| 47887 | Freidreich's ataxia |
| 47888 | Freidreichs ataxia |
| 47889 | Freidriech ataxia |
| 47890 | Freidriech's ataxia |
| 47891 | Freidriechs ataxia |
| 47892 | Friedreich ataxia |
| 47893 | Friedreich's, ataxia |
| 47894 | Friedreichs ataxia |
| 47895 | Friedriech ataxia |
| 47896 | Friedriech's ataxia |
| 47897 | Friedriechs ataxia |
| 47898 | Hereditary ataxia of Friedreich |
| 47899 | Hereditary spinal ataxia |
| 47900 | Hereditary spinal scleroses |
| 47901 | Hereditary spinal sclerosis |
| 47903 | Sclerosis, hereditary spinal |
| 47904 | Sclerosis, hereditary, spinal |
| 47905 | Sclerosis, spinal, hereditary |
| 47906 | Spinal scleroses, hereditary |
| 47907 | Spinal sclerosis, hereditary |
| 48827 | Syndrome, pellagra-cerebellar ataxia-renal aminoaciduria |
| 54756 | Degeneration, cerebellar, primary |
| 64666 | Ataxia, hereditary NEC |
| 65244 | Hereditary areflexic dystasia |
| 68903 | Ataxia, cerebellar, hereditary |
| 68904 | Ataxia, hereditary, cerebellar |
| 68905 | Ataxia, Marie's |
| 68906 | Familial cerebellar ataxia |
| 68907 | Familial cerebellar degeneration syndrome |
| 68908 | Marie's ataxia |
| 73168 | Hereditary primary cerebellar degeneration |
| 73170 | Ataxia-telangiectasia (Louis-Bar syndrome) |
| 78880 | Familial ataxia |
| 93887 | Friedreich's ataxia |
| 96485 | Ataxia, familial, spinal |
| 96486 | Ataxia, family, spinal |
| 96487 | Ataxia, Friedreich's heredofamilial |
| 96488 | Ataxia, Friedreich's spinal |
| 96489 | Ataxy, familial, spinal |
| 96490 | Ataxy, family, spinal |
| 96491 | Ataxy, Friedreich's heredofamilial |
| 96492 | Ataxy, Friedreich's spinal |
| 98464 | FA (Friedreich ataxia) |
| 98511 | Family ataxia |
| 98780 | Friedreich's tabes |
| 98781 | Friedreich's, sclerosis |
| 98782 | Friedrich's ataxia |
| 98783 | Friedrick's ataxia |
| 101993 | Sclerosis, Friedreich's |
| 102295 | Spinal sclerosis hereditary |
| 102300 | Spino-cerebellar degeneration |
| 102302 | Spinocerebellar degeneration |
| 102304 | Spinocerebellar disorder |
| 104263 | Ataxia telangiectasia |
| 104266 | Ataxia, hereditary |
| 104267 | Ataxia, Marie's cerebellar |
| 104268 | Ataxia-telangiectasias |
| 104269 | Ataxias, hereditary |
| 104482 | Behr's syndrome |
| 106985 | Disease, Freidreich |
| 109310 | Hereditary ataxias |
| 111238 | Marie's, cerebellar ataxia |
| 116563 | Cerebellar ataxia /dyskinesia |
| 119318 | Hereditary ataxia, Friedreich's |
| 119319 | Hereditary ataxia, Friedreich's |
| 119320 | Familial ataxia, Friedreich's |
| 119321 | Familial ataxia, Friedreich's |
| 119322 | Friedreich's hereditary spinal ataxia |
| 119323 | Friedreich's hereditary spinal ataxia |
| 119324 | Hereditary ataxia, Friedreich |
| 119325 | Hereditary ataxia, Friedreich |
| 119326 | Ataxia, Friedreich spinocerebellar |
| 119327 | Ataxia, Friedreich spinocerebellar |
| 119328 | Ataxia, Friedreich hereditary |
| 119329 | Ataxia, Friedreich hereditary |
| 119330 | Friedreichs familial ataxia |
| 119331 | Friedreichs familial ataxia |
| 119332 | Friedreich familial ataxia |
| 119333 | Friedreich familial ataxia |
| 119334 | Friedreich's hereditary ataxia |
| 119335 | Friedreich's hereditary ataxia |
| 119336 | Friedreich's familial ataxia |
| 119337 | Friedreich's familial ataxia |
| 119338 | Familial ataxia, Friedreich |
| 119339 | Familial ataxia, Friedreich |
| 119340 | Friedreich hereditary spinal ataxia |
| 119341 | Friedreich hereditary spinal ataxia |
| 119342 | Friedreich hereditary ataxia |
| 119343 | Friedreich hereditary ataxia |
| 119344 | Friedreich spinocerebellar ataxia |
| 119345 | Friedreich spinocerebellar ataxia |
| 119346 | Spinocerebellar ataxia, Friedreich |
| 119347 | Spinocerebellar ataxia, Friedreich |
| 121699 | Polyneuritiformi, hemeralopia heredoataxia |
| 121921 | Cerebellar ataxia, Marie's |
| 121924 | Cerebellar ataxia, Marie |
| 125211 | Ataxia telangiectasia syndrome |
| 127650 | Syndrome, ataxia telangiectasia |
| 128836 | Truncal ataxia |
| 128933 | Ataxia, Friedreich familial |
| 128934 | Ataxia, Friedreich's familial |
| 128935 | Ataxia, Friedreich's hereditary |
| 128936 | Hereditary spinal ataxia, Friedreich |
| 128937 | Hereditary spinal ataxia, Friedreich's |
| 128942 | Ataxia, Friedreich familial |
| 128943 | Ataxia, Friedreich's familial |
| 128944 | Ataxia, Friedreich's hereditary |
| 128945 | Corticostriatal-spinal degenerations |
| 128946 | Degeneration, hereditary spinocerebellar |
| 128947 | Degeneration, inherited spinocerebellar |
| 128948 | Familial spinocerebellar degeneration |
| 128949 | Familial spinocerebellar degenerations |
| 128950 | Hereditary spinal ataxia, Friedreich |
| 128951 | Hereditary spinal ataxia, Friedreich's |
| 128952 | Hereditary spinocerebellar degeneration |
| 128977 | Ataxia, Friedreich familial |
| 128978 | Ataxia, Friedreich's familial |
| 128979 | Ataxia, Friedreich's hereditary |
| 129005 | Hereditary spinal ataxia, Friedreich |
| 129006 | Hereditary spinal ataxia, Friedreich's |
| 129061 | Familial spinocerebellar degeneration |
| 129063 | Hereditary spinocerebellar degeneration |
| 131154 | Appendicular ataxia |
| 132897 | Machado-Joseph disease |
| 135701 | Holmes's cerebellar degeneration |
| 136718 | Ataxia with lactic acidosis I |
| 136721 | Ataxia with lactic acidosis II |
| 140774 | Neuropathy, ataxia and retinitis pigmentosa |
| 140775 | NARP (neuropathy, ataxia and retinitis pigmentosa) |
| 140776 | NARP syndrome (neuropathy, ataxia and retinitis pigmentosa) |
| 143953 | SCA-1 (spinocerebellar ataxia type 1) |
| 143954 | SCA-2 (spinocerebellar ataxia type 2) |
| 143955 | SCA-3 (spinocerebellar ataxia type 3) |
| 143956 | SCA-4 (spinocerebellar ataxia type 4) |
| 143957 | SCA-5 (spinocerebellar ataxia type 5) |
| 143958 | SCA-6 (spinocerebellar ataxia type 6) |
| 143959 | SCA-7 (spinocerebellar ataxia type 7) |
| 143960 | SCA-8 (spinocerebellar ataxia type 8) |
| 143961 | SCA-10 (spinocerebellar ataxia type 10) |
| 143962 | SCA-11 (spinocerebellar ataxia type 11) |
| 143963 | SCA-12 (spinocerebellar ataxia type 12) |
| 143986 | Spinocerebellar ataxia |
| 147835 | CACH (childhood ataxia with central hypomyelination syndrome) |
| 148833 | Cerebellar ataxia associated with another disorder |
| 150754 | Ataxia with lactic acidosis I |
| 150816 | Maries cerebellar ataxia |
| 150817 | Marie cerebellar ataxia |
| 150818 | Cerebellar ataxia, Marie's |
| 150819 | Cerebellar ataxia, Marie |
| 150903 | Friedriech's disease |
| 150904 | Friedriechs disease |
| 150905 | Friedriech disease |
| 150906 | Friedreich's, disease |
| 150907 | Friedreichs disease |
| 150908 | Friedreich disease |
| 150909 | Freidreich's disease |
| 150910 | Freidreichs disease |
| 150911 | Freidreich disease |
| 150912 | Disease, Friedriech's |
| 150913 | Disease, Friedriech |
| 150914 | Disease, Friedreich's |
| 150915 | Disease, Friedreich |
| 150916 | Disease, Freidreich's |
| 162250 | Neuropathy in association with hereditary ataxia |
| 169360 | Neuropathy in association with hereditary ataxia |
| 177639 | Episodic ataxia |
| 177651 | Ataxia with oculomotor apraxia |
| 177652 | Ataxia with oculomotor apraxia type 2 |
| 182611 | Episodic ataxia |
| 182773 | Ataxia with oculomotor apraxia type 1 |
| 193430 | Hereditary ataxia, unspecified |
| 197944 | Hereditary ataxia unspecified |
| 198282 | Neuropathy ass with hereditary ataxia |
| 206679 | Neuropathy, ataxia, and retinitis pigmentosa |
| 226716 | Early-onset cerebellar ataxia |
| 227142 | Hunt's striatal syndrome (1) |
| 227143 | Hunt's striatal syndrome (2) |
| 231561 | Ataxia, hereditary |
| 231562 | Ataxias, hereditary |
| 231563 | Family ataxia |
| 231564 | Hereditary ataxia |
| 231565 | Hereditary ataxias |
| 231566 | Hereditary ataxia, unspecified |
| 231807 | Hypertrophic cardiomyopathy secondary to Friedreich's ataxia |
| 253421 | Hereditary spastic ataxia |
| 253422 | Spastic ataxia, hereditary |
| 308210 | Cerebellar ataxia with defective DNA repair |
| 308377 | Congenital nonprogressive ataxia |
| 329128 | Mitochondrial ataxia syndrome |
| 329129 | Mitochondrial disorder with ataxia |
| 339264 | Childhood ataxia with diffuse central nervous system hypomyelination syndrome |
| 376643 | Dyssynergia cerebellaris myoclonica of Ramsay Hunt |
| 379333 | Neurodegenerative disease with dementia, ataxia, and spasticity |
| 379334 | Neurodegenerative disease with dementia, ataxia, and spasticity |
| 379335 | Neurodegenerative disease with dementia, ataxia, and spasticity |
| 380541 | Ataxia, hereditary, spinal |
| 380542 | Ataxia, spinal, hereditary |
| 380545 | Hereditary spinal ataxia |
| 417880 | Ataxia, cataracts, retarded somatic and mental maturation |
| 428929 | Autosomal dominant cerebellar ataxia, deafness, and narcolepsy |
| 485845 | Mild autosomal dominant cerebellar ataxia, deafness, and narcolepsy |
| 485846 | Moderate autosomal dominant cerebellar ataxia, deafness, and narcolepsy |
| 485847 | Severe autosomal dominant cerebellar ataxia, deafness, and narcolepsy |
| 487995 | Autosomal dominant cerebellar ataxia, deafness, and narcolepsy, mild |
| 488073 | Autosomal dominant cerebellar ataxia, deafness, and narcolepsy, severe |
| 488341 | Autosomal dominant cerebellar ataxia, deafness, and narcolepsy, moderate |
| 505782 | Autosomal recessive spastic ataxia type 2 |
| 505866 | Spinocerebellar ataxia type 23 |
| 505868 | Spinocerebellar ataxia type 4 |
| 505913 | Spinocerebellar ataxia type 8 |
| 505914 | Spinocerebellar ataxia type 5 |
| 505936 | Spinocerebellar ataxia type 6 |
| 505956 | Autosomal recessive spinocerebellar ataxia type 17 |
| 505972 | Autosomal recessive spinocerebellar ataxia type 11 |
| 506051 | Spinocerebellar ataxia type 28 |
| 506054 | Autosomal recessive spinocerebellar ataxia type 10 |
| 506104 | Spinocerebellar ataxia type 26 |
| 506111 | Autosomal recessive spinocerebellar ataxia type 7 |
| 506160 | Posterior column ataxia with retinitis pigmentosa |
| 506165 | Autosomal recessive spinocerebellar ataxia type 13 |
| 506166 | Spinocerebellar ataxia type 10 |
| 506177 | Spinocerebellar ataxia type 29 |
| 506227 | Spinocerebellar ataxia type 36 |
| 506305 | Spinocerebellar ataxia type 31 |
| 506318 | Spinocerebellar ataxia type 20 |
| 506349 | Autosomal dominant cerebellar ataxia linked to chromosome 16q |
| 506354 | Autosomal recessive spastic ataxia type 5 |
| 506358 | Spinocerebellar ataxia type 34 |
| 506363 | Autosomal dominant sensory ataxia type 1 |
| 506399 | Spinocerebellar ataxia type 25 |
| 506450 | Ataxia neuropathy spectrum associated with mutation in POLG gene |
| 506456 | Spinocerebellar ataxia type 35 |
| 506489 | Spinocerebellar ataxia type 19 |
| 506540 | Spinocerebellar ataxia type 3 |
| 506573 | Late onset Friedreich ataxia |
| 506599 | Autosomal recessive spastic ataxia type |
| 506603 | POLG-related ataxia neuropathy spectrum |
| 506615 | Spinocerebellar ataxia type 18 |
| 506733 | Spinocerebellar ataxia type 21 |
| 510206 | Episodic ataxia type 4 |
| 510277 | Episodic ataxia type 7 |
| 510326 | Episodic ataxia type 3 |
| 515067 | Spinocerebellar ataxia type 12 |
| 515132 | Spinocerebellar ataxia type 7 |
| 515143 | Cuban type spinocerebellar ataxia |
| 515147 | Childhood onset spinocerebellar ataxia type 13 |
| 515158 | Autosomal recessive spinocerebellar ataxia type 1 |
| 515163 | Spinocerebellar ataxia type 2 |
| 515167 | Autosomal dominant spinocerebellar ataxia type 17 |
| 515219 | Spinocerebellar ataxia type 15 |
| 515264 | Spinocerebellar ataxia type 11 |
| 515291 | Adult onset spinocerebellar ataxia type 13 |
| 515307 | Adult onset spinocerebellar ataxia type 1 |
| 515328 | Juvenile onset spinocerebellar ataxia type 1 |
| 515331 | Spinocerebellar ataxia type 14 |
| 515354 | Spinocerebellar ataxia with axonal neuropathy |
| 515446 | Autosomal recessive spastic ataxia type 3 |
| 515459 | Ataxia-telangiectasia-like disorder |
| 515551 | X-linked spinocerebellar ataxia type 4 |
| 515765 | Autosomal recessive Charlevoix-Saguenay type spastic ataxia |
| 515770 | SYNE1-related spinocerebellar ataxia type 8, autosomal recessive |
| 515814 | Spinocerebellar ataxia with blindness and deafness |
| 515815 | X-linked spinocerebellar ataxia type 3 |
| 515965 | Cayman type cerebellar ataxia |
| 516383 | Muscular atrophy, ataxia, retinitis pigmentosa, and diabetes mellitus syndrome |
| 520571 | PRICKLE1-related progressive myoclonus epilepsy with ataxia |
| 520614 | X-linked ataxia, apraxia, and mental retardation |
| 520711 | Progressive myoclonus epilepsy with ataxia associated with mutation in PRICKLE1 gene |
| 521625 | Cerebellar vermis hypoplasia, oligophrenia, congenital ataxia, ocular coloboma, and hepatic fibrosis syndrome |
| 521656 | Nonprogressive cerebellar ataxia with intellectual disability |
| 523994 | Autosomal recessive spinocerebellar atrophy type 5 |
| 524018 | Cerebellar ataxia, mental retardation, optic atrophy, and skin abnormalities (CAMOS) syndrome |
| 524141 | Autosomal recessive spinocerebellar atrophy type 4 |
| 524285 | Spinocerebellar ataxia with saccadic intrusions |
| 525683 | Fragile X associated tremor ataxia syndrome |
| 525817 | Spinocerebellar degeneration, macular corneal dystrophy, congenital cataracts, and myopia syndrome |
| 526003 | Early onset cerebellar ataxia with retained reflexes |
| 526087 | Spinocerebellar atrophy type 3 subtype 2 |
| 526115 | Machado-Joseph disease type 2 |
| 526169 | Machado-Joseph disease type 1 |
| 526349 | Spinocerebellar atrophy type 3 subtype 1 |
| 526430 | Infantile nonprogressive spinocerebellar ataxia type 6 |
| 526440 | Autosomal recessive spinocerebellar ataxia type 6 |
| 527581 | X-linked ataxia dementia syndrome |
| 527708 | Gordon-Holmes syndrome associated with mutation in PNPLA6 gene |
| 527709 | PNPLA6-related Gordon-Holmes syndrome |
| 527710 | Gordon-Holmes syndrome associated with mutation in RNF216 gene |
| 527711 | RNF216-related Gordon-Holmes syndrome |
| 527789 | Episodic ataxia associated with mutation in KCNA1 gene |
| 527803 | X-linked ataxia, apraxia, and intellectual disability |
| 527818 | Episodic ataxia type 1 |
| 527819 | Episodic ataxia associated with mutation in CACNA1A gene |
| 527820 | Episodic ataxia type 2 |
| 527821 | Episodic ataxia associated with mutation in CACNB4 |
| 527822 | Episodic ataxia type 5 |
| 527823 | Episodic ataxia associated with mutation in SLC1A3 gene |
| 527824 | Episodic ataxia type 6 |
| 528399 | Ketoaciduria, intellectual disability, ataxia, and deafness syndrome |
| 528523 | Ataxia telangiectasia mutated (ATM) protein in undetectable to low range |
| 528524 | Ataxia, deafness, and intellectual disability syndrome |
| 528525 | Ataxia, deafness, and mental retardation syndrome |
| 528526 | Ataxia oculomotor apraxia type 3 |
| 528527 | Ataxia-oculomotor apraxia associated with mutation in PIK3R5 |
| 528528 | Autosomal recessive ataxia oculomotor apraxia type 2 |
| 528711 | Spinocerebellar ataxia associated with mutation in FGF14 gene |
| 528712 | Spinocerebellar ataxia type 27 |
| 528713 | X-linked spinocerebellar ataxia type 5 |
| 528714 | Autosomal recessive spinocerebellar ataxia associated with mutation in WWOX gene |
| 528715 | Autosomal recessive spinocerebellar ataxia type 12 |
| 529061 | X-linked congenital spinocerebellar ataxia type 1 |
| 529064 | Polyneuropathy, hearing loss, ataxia, retinitis pigmentosa, and cataract syndrome |
| 529086 | Seizures, sensorineural deafness, ataxia, intellectual disability, and electrolyte imbalance syndrome |
| 530721 | Autosomal recessive spinocerebellar ataxia associated with mutation in SYNE1 gene |
| 530722 | Autosomal recessive spinocerebellar ataxia type 8 |
| 531141 | 16q-linked autosomal dominant cerebellar ataxia |
| 531179 | Ataxia pancytopenia syndrome |
| 531737 | Myoclonic epilepsy, myopathy, and sensory ataxia (MEMSA) associated with mutation in POLG gene |
| 531738 | POLG-related myoclonic epilepsy, myopathy, and sensory ataxia (MEMSA) |
| 531740 | Myoclonus, cerebellar ataxia, and deafness syndrome |
| 532288 | Autosomal dominant spastic ataxia type 1 |
| 532735 | Autosomal recessive spinocerebellar ataxia associated with mutation in SPTBN2 gene |
| 532736 | Autosomal recessive spinocerebellar ataxia type 14 |
| 532737 | Autosomal recessive spinocerebellar ataxia associated with mutation in KIAA0226 gene |
| 532738 | Autosomal recessive spinocerebellar ataxia type 15 |
| 532739 | Autosomal recessive spinocerebellar ataxia associated with mutation in STUB1 gene |
| 532740 | Autosomal recessive spinocerebellar ataxia type 16 |
| 533042 | Paroxysmal choreoathetosis with episodic ataxia and spasticity |
| 534268 | Hereditary ataxia |
| 538507 | Neuropathy in association with hereditary ataxia |
| 539064 | Early-onset cerebellar ataxia |
| 539532 | Hereditary ataxia, unspecified |
| 607053 | Cerebellar ataxia with defective DNA repair |
| 608629 | Other hereditary ataxias |
| 1105234 | Neuropathy associated with hereditary ataxia |
| 1111271 | Ataxia-telangiectasia-like disorder associated with mutation in PCNA gene |
| 1111278 | Ataxia-telangiectasia-like disorder type 2 |
| 1111586 | Spinocerebellar ataxia type 38 |
| 1111591 | Spinocerebellar ataxia associated with mutation in ELOVL5 gene |
| 1111973 | Spinocerebellar ataxia type 40 |
| 1112136 | Ataxia, combined cerebellar and peripheral, with hearing loss and diabetes mellitus |
| 1112198 | Spinocerebellar ataxia associated with mutation in CCDC88C gene |
| 1112333 | Autosomal recessive spinocerebellar ataxia type 18 |
| 1112366 | Autosomal recessive spinocerebellar ataxia associated with mutation in GRID2 gene |
| 1112392 | Ataxia-oculomotor apraxia type 4 |
| 1112420 | Autosomal recessive spinocerebellar ataxia associated with mutation in CWF19L1 gene |
| 1112478 | Ataxia with oculomotor apraxia associated with mutation in PNKP gene |
| 1113963 | Ataxia due to recent cerebrovascular accident |
| 1137812 | Hereditary ataxia, unspecified |
| 1192243 | Other hereditary ataxias |
| 1273939 | Spinocerebellar ataxia type 1 |
| 1274103 | Paroxysmal dystonic choreoathetosis with episodic ataxia and spasticity |
| 1274355 | Spinocerebellar ataxia type 15/16 |
| 1274363 | Cerebellar ataxia with ectodermal dysplasia |
| 1274412 | Cerebellar ataxia with hypogonadism and choroidal dystrophy syndrome |
| 1274442 | Autosomal recessive cerebelloparenchymal disorder type 3 |
| 1299930 | Cardiomyopathy associated with Friedreich ataxia |
| 1299934 | Cardiomyopathy associated with Friedreich's ataxia |
| 1348384 | ATP8A2-related cerebellar ataxia, mental retardation, and dysequilibrium syndrome |
| 1348385 | Cerebellar ataxia, intellectual disability, and dysequilibrium syndrome associated with mutation in WDR81 gene |
| 1348387 | X-linked sideroblastic anemia and ataxia syndrome in female |
| 1348416 | Cerebellar ataxia, intellectual disability, and dysequilibrium syndrome associated with mutation in CA8 gene |
| 1348418 | CA8-related cerebellar ataxia, mental retardation, and dysequilibrium syndrome |
| 1348422 | Cerebellar ataxia, areflexia, pes cavus, optic atrophy, and sensorineural hearing loss syndrome |
| 1348448 | WDR81-related cerebellar ataxia, mental retardation, and dysequilibrium syndrome |
| 1348449 | Cerebellar ataxia, intellectual disability, and dysequilibrium syndrome associated with mutation in ATP8A2 gene |
| 1348991 | X-linked sideroblastic anemia with spinocerebellar ataxia |
| 1349438 | Ataxia due to mitochondrial mutations |
| 1349878 | Congenital cataract, ataxia, and deafness syndrome |
| 1350262 | Spinocerebellar ataxia type 37 |
| 1350395 | Spinocerebellar ataxia type 17 |
| 1350414 | Spinocerebellar ataxia type 32 |
| 1350471 | Spinocerebellar ataxia type 30 |
| 1350598 | Spinocerebellar ataxia type 13 |
| 1351460 | Congenital cataract-ataxia-deafness syndrome |
| 1352386 | Autosomal recessive spinocerebellar ataxia associated with mutation in RUBCN gene |
| 1352541 | Autosomal recessive spinocerebellar ataxia type 9 |
| 1352704 | Cataract with ataxia, short stature, and intellectual disability |
| 1352788 | Sideroblastic anemia with spinocerebellar ataxia |
| 1352850 | Cataract with ataxia and deafness |
| 1352943 | Myoclonus with cerebellar ataxia and deafness |
| 1353288 | Dilated cardiomyopathy with ataxia syndrome |
| 1353428 | Cerebellar ataxia and hypogonadotropic hypogonadism |
| 1353476 | Spinocerebellar ataxia type 9 |
| 1353624 | Autosomal recessive spinocerebellar ataxia type 5 |
| 1353661 | Autosomal recessive spinocerebellar ataxia type 4 |
| 1353736 | Early onset ataxia with oculomotor apraxia and hypoalbuminemia |
| 1353962 | X-linked spinocerebellar ataxia type 2 |
| 1353996 | Autosomal recessive spinocerebellar ataxia type 3 |
| 1354307 | Autosomal recessive spinocerebellar ataxia with axonal neuropathy |
| 1354388 | Renal dysplasia with retinal pigmentary dystrophy, cerebellar ataxia, and skeletal dysplasia |
| 1355197 | Deafness with hyperuricemia and neurologic ataxia |
| 1355511 | Infantile onset spinocerebellar ataxia |
| 1355644 | Hypotonia with congenital nystagmus, ataxia, and abnormal brainstem auditory response |
| 1430097 | Ataxia due to abetalipoproteinemia |
| 1430198 | Ataxia due to cerebrotendinous xanthomatosis |
| 1430232 | Ataxia due to Refsum's disease |
| 1430619 | Autosomal recessive posterior column ataxia with retinitis pigmentosa |
| 1432108 | Ataxia due to phytanic acid storage disease |
| 1445740 | X-linked nonprogressive cerebellar ataxia |
| 1445751 | Autosomal recessive cerebellar ataxia, pyramidal signs, nystagmus, and oculomotor apraxia syndrome |
| 1445803 | Autosomal recessive cerebellar ataxia and saccadic intrusion syndrome |
| 1445968 | Autosomal recessive cerebellar ataxia with late onset spasticity |
| 1446016 | Cerebellar ataxia, intellectual disability, oculomotor apraxia, and cerebellar cysts syndrome |
| 1446026 | Benign paroxysmal tonic upgaze of childhood with ataxia |
| 1446045 | Ataxia telangiectasia variant |
| 1449808 | Episodic ataxia with slurred speech |
| 1450217 | Early onset progressive neurodegeneration, blindness, ataxia, and spasticity syndrome |
| 1450248 | Early onset spastic ataxia, myoclonic epilepsy, and neuropathy syndrome |
| 1450576 | Ataxia, photosensitivity, and short stature syndrome |
| 1450736 | Autosomal recessive cerebellar ataxia, epilepsy, and intellectual disability syndrome due to WW domain-containing oxidoreductase deficiency |
| 1458189 | Heterozygous autosomal recessive spinocerebellar ataxia type 1 |
| 1458190 | Heterozygous ataxia oculomotor apraxia type 2 |
| 1458569 | Recessive mitochondrial ataxia syndrome |
| 1458605 | Adult-onset autosomal recessive cerebellar ataxia |
| 1459250 | Intellectual disability, hyperkinetic movement, and truncal ataxia syndrome |
| 1459349 | Hypotonia, ataxia, and developmental delay syndrome (HADDS) |
| 1459912 | Adult onset autosomal recessive cerebellar ataxia |
| 1460008 | Childhood onset autosomal recessive slowly progressive spinocerebellar ataxia |
| 1462309 | X-linked progressive cerebellar ataxia |
| 1464211 | Friedreich ataxia |
| 1465810 | Early-onset cerebellar ataxia, unspecified |
| 1465978 | Other early-onset cerebellar ataxia |
| 1466772 | Progressive spinal ataxia |
| 1466832 | Myoclonic epilepsy myopathy sensory ataxia |
| 1470096 | Renal dysplasia-retinal pigmentary dystrophy-cerebellar ataxia-skeletal dysplasia syndrome |
| 1470283 | Spinocerebellar ataxia with epilepsy |
| 1473326 | Progressive myoclonic epilepsy with ataxia |
| 1477574 | Autosomal dominant cerebellar ataxia type 2 |
| 1479119 | Other hereditary ataxias |
| 1479694 | Congenital cerebellar ataxia due to mutation in RNU12 gene |
| 1479773 | Autosomal recessive spinocerebellar ataxia type 21 |
| 1479982 | Early-onset progressive encephalopathy, spastic ataxia, and distal spinal muscular atrophy syndrome |
| 1485036 | Cerebellar ataxia with oculomotor apraxia type 4 |
| 1485192 | Spinocerebellar ataxia type 41 |
| 1485233 | Spinocerebellar ataxia type 42 |
| 1485335 | Spinocerebellar ataxia type 43 |
| 1485387 | Autosomal recessive spinocerebellar ataxia, blindness, and deafness syndrome |
| 1485427 | Optic atrophy, ataxia, peripheral neuropathy, and global developmental delay syndrome |
| 1485793 | Severe intellectual disability, agenesis of corpus callosum, facial dysmorphism, and cerebellar ataxia syndrome |
| D64.0 | X-linked sideroblastic anemia with spinocerebellar ataxia |
| D64.3 | X-linked sideroblastic anemia with spinocerebellar ataxia |
| E71.111 | Dilated cardiomyopathy with ataxia syndrome |
| E74.4 | Ataxia with lactic acidosis I |
| E75.29 | CACH (childhood ataxia with central hypomyelination syndrome) |
| E75.5 | Ataxia due to cerebrotendinous xanthomatosis |
| E78.6 | Ataxia due to abetalipoproteinemia |
| E87.2 | Ataxia with lactic acidosis II |
| E87.20 | Ataxia with lactic acidosis II |
| E88.49 | Neuropathy, ataxia and retinitis pigmentosa |
| G11.0 | Congenital nonprogressive ataxia |
| G11.1 | Friedreich's ataxia |
| G11.10 | Early-onset cerebellar ataxia |
| G11.11 | Friedreich's ataxia |
| G11.19 | Hunt's striatal syndrome (1) |
| G11.2 | Marie's cerebellar ataxia |
| G11.3 | Ataxia-telangiectasia syndrome |
| G11.4 | Hereditary spastic ataxia |
| G11.8 | Primary cerebellar degeneration |
| G11.9 | Cerebellar ataxia |
| G31.89 | X-linked ataxia, apraxia, and mental retardation |
| G32.81 | Cerebellar ataxia in diseases classified elsewhere |
| G40.409 | PRICKLE1-related progressive myoclonus epilepsy with ataxia |
| G60.0 | Hereditary ataxia-muscular atrophy syndrome |
| G60.1 | Ataxia due to Refsum's disease |
| G60.2 | Neuropathy in association with hereditary ataxia |
| G96.89 | Ataxia pancytopenia syndrome |
| G98.8 | Ketoaciduria, intellectual disability, ataxia, and deafness syndrome |
| H26.9 | Cataract with ataxia, short stature, and intellectual disability |
| H35.52 | Posterior column ataxia with retinitis pigmentosa |
| H51.8 | Ataxia with oculomotor apraxia |
| H55.01 | Ataxia oculomotor apraxia type 3 |
| Q87.89 | Ataxia, deafness, and intellectual disability syndrome |
| Q99.2 | Fragile X associated tremor ataxia syndrome |
| Q99.9 | Autosomal dominant cerebellar ataxia linked to chromosome 16q (HCC) |
| R27.8 | Hereditary ataxia-muscular atrophy syndrome |
| 251026 | Hereditary ataxia, unspecified |
| 259572 | Hereditary ataxia, unspecified |
| 52 | Ataxia following cerebral infarction |
| 162.9 | Malignant neoplasm of bronchus and lung, unspecified |
| 239.9 | Neoplasm of unspecified nature, site unspecified |
| 249.6 | Other specified diabetes mellitus with diabetic mononeuropathy |
| 269.1 | Deficiency of other vitamins |
| 303.9 | Other and unspecified alcohol dependence, unspecified |
| 337.1 | Type 2 diabetes mellitus with diabetic neuropathy, unspecified |
| 436 | Other cerebrovascular disease |
| 437.9 | Cerebrovascular disease, unspecified |
| 438.84 | Ataxia following nontraumatic intracerebral haemorrhage |
| 438.9 | Ataxia following cerebral infarction |
| 908.9 | Late effect of unspecified injury |
| 16202 | Other late effects of cerebrovascular disease, ataxia |
| 36756 | Postvaricella encephalitis |
| 131157 | Unspecified disorder of muscle, ligament, and fascia |
| 131741 | Lack of coordination |
| 149430 | Other late effects of cerebrovascular disease, ataxia |
| 149432 | Other late effects of cerebrovascular disease, ataxia |
| 149437 | Other late effects of cerebrovascular disease, ataxia |
| 149438 | Other late effects of cerebrovascular disease, ataxia |
| 156286 | Ataxia following unspecified cerebrovascular disease |
| 156725 | Other late effects of cerebrovascular disease, ataxia |
| 161464 | Unspecified cerebrovascular disease |
| 161465 | Unspecified cerebrovascular disease |
| 161466 | Unspecified cerebrovascular disease |
| 161467 | Unspecified cerebrovascular disease |
| 177640 | Deficiency of other vitamins |
| 177641 | Deficiency of other vitamins |
| 182774 | Lack of coordination |
| 187478 | Type 2 diabetes mellitus with diabetic mononeuropathy |
| 187480 | Peripheral autonomic neuropathy in disorders classified elsewhere |
| 187481 | Type 2 diabetes mellitus with diabetic mononeuropathy |
| 187482 | Peripheral autonomic neuropathy in disorders classified elsewhere |
| 187484 | Secondary diabetes mellitus with neurological manifestations, not stated as uncontrolled, or unspecified |
| 187485 | Other specified diabetes mellitus with diabetic mononeuropathy |
| 202747 | Unspecified cerebrovascular disease |
| 202748 | Lack of coordination |
| 202749 | Ataxia, unspecified |
| 246407 | Other late effects of cerebrovascular disease, ataxia |
| 252181 | Other cerebellar ataxia |
| 252182 | Ataxia following cerebral infarction |
| 252183 | Other late effects of cerebrovascular disease, ataxia |
| 252184 | Ataxia following cerebral infarction |
| 252185 | Other late effects of cerebrovascular disease, ataxia |
| 252186 | Ataxia following cerebral infarction |
| 262569 | Other late effects of cerebrovascular disease, ataxia |
| 267965 | Unspecified late effects of cerebrovascular disease |
| 267966 | Other late effects of cerebrovascular disease, ataxia |
| 267967 | Other late effects of cerebrovascular disease, ataxia |
| 267968 | Other late effects of cerebrovascular disease, ataxia |
| 267969 | Other late effects of cerebrovascular disease, ataxia |
| 290339 | Ataxia following unspecified cerebrovascular disease |
| 317237 | Ataxia following cerebral infarction |
| 317238 | Lack of coordination |
| 317239 | Lack of coordination |
| 383010 | Ataxia following cerebral infarction |
| 383011 | Ataxia following cerebral infarction |
| 383232 | Other late effects of cerebrovascular disease, ataxia |
| 383300 | Other and unspecified alcohol dependence, unspecified |
| 383685 | Other late effects of cerebrovascular disease, ataxia |
| 383695 | Ataxia, unspecified |
| 383782 | Late effect of unspecified injury |
| 383803 | Cerebellar ataxia in diseases classified elsewhere |
| 383964 | Late effect of unspecified injury |
| 384561 | Ataxia following unspecified cerebrovascular disease |
| 401289 | Ataxia following nontraumatic intracerebral haemorrhage |
| 401293 | Other late effects of cerebrovascular disease, ataxia |
| 402244 | Other late effects of cerebrovascular disease, ataxia |
| 402245 | Other late effects of cerebrovascular disease, ataxia |
| 493048 | Alcohol dependence, uncomplicated |
| 523965 | Other cerebellar ataxia |
| 526073 | Malignant neoplasm of bronchus and lung, unspecified |
| 589026 | Ataxia following other cerebrovascular disease |
| 589513 | Ataxia following nontraumatic intracerebral hemorrhage |
| 589514 | Ataxia following nontraumatic subarachnoid hemorrhage |
| 589515 | Ataxia following cerebral infarction |
| 592386 | Ataxia following other nontraumatic intracranial hemorrhage |
| 598711 | Ataxia following unspecified cerebrovascular disease |
| 632541 | Ataxia following other cerebrovascular disease |
| 632963 | Ataxia following other nontraumatic intracranial hemorrhage |
| 1113951 | Ataxia following cerebral infarction |
| 1113963 | Ataxia following cerebral infarction |
| 1182290 | Ataxia following unspecified cerebrovascular disease |
| 1317876 | Other cerebellar ataxia |
| 1319052 | Toxic effect of unspecified substance, accidental (unintentional), initial encounter |
| 1431317 | Ataxia following nontraumatic subarachnoid hemorrhage |
| 1431384 | Other late effects of cerebrovascular disease, ataxia |
| 1439528 | Ataxia following cerebral infarction |
| 1439661 | Ataxia following nontraumatic intracerebral hemorrhage |
| 1440016 | Other late effects of cerebrovascular disease, ataxia |
| 1450082 | Ataxia following cerebral infarction |
| 1478868 | Ataxia, unspecified |
| 1478939 | Lack of coordination |
| C34.90 | Cerebellar ataxia associated with malignant neoplasm of lung |
| I67.9 | Ataxia, unspecified |
| I69.093 | Ataxia following nontraumatic subarachnoid hemorrhage |
| I69.193 | Ataxia following nontraumatic intracerebral hemorrhage |
| I69.293 | Ataxia following other nontraumatic intracranial hemorrhage |
| I69.393 | Ataxia following cerebral infarction |
| I69.893 | Ataxia following other cerebrovascular disease |
| I69.993 | Ataxia following other nontraumatic intracranial hemorrhage |
| 182775 | AVED (ataxia with vitamin E deficiency) |
| R27.0 | AVED (ataxia with vitamin E deficiency) |
| 315.4 | Developmental coordination disorder |
| 334.4 | Cerebellar ataxia in diseases classified elsewhere |
| 572.8 | Ataxias, sensory |
| 728.85 | Ataxia muscularis |
| 728.9 | Appendicular ataxia |
| 781.3 | Ataxia of both legs |
| 3463 | Cerebellar ataxia in diseases classified elsewhere |
| 30719 | Cerebral ataxia |
| 30904 | Cerebral paresis with homolateral ataxia |
| 43407 | Ataxia, unspecified |
| 43408 | Ataxia, unspecified |
| 44452 | Other cerebellar ataxia |
| 44454 | Other cerebellar ataxia |
| 44455 | Hereditary ataxia, unspecified |
| 44456 | Other cerebellar ataxia |
| 53681 | Other cerebellar ataxia |
| 56982 | Other specified neurosyphilis |
| 70657 | Locomotor ataxia (progressive) |
| 73169 | Other hereditary ataxias |
| 78037 | Lack of coordination |
| 83887 | Other cerebellar ataxia |
| 92823 | Cerebellar ataxia in diseases classified elsewhere |
| 104264 | Other cerebellar ataxia |
| 104265 | Other cerebral degeneration |
| 118425 | Other cerebellar ataxia |
| 118427 | Other cerebellar ataxia |
| 118431 | Other cerebellar ataxia |
| 118432 | Cerebellar ataxia in diseases classified elsewhere |
| 118433 | Other cerebellar ataxia |
| 118435 | Other cerebellar ataxia |
| 121923 | Spinocerebellar disease, unspecified |
| 123586 | Ataxia, unspecified |
| 128808 | Developmental coordination disorder |
| 128809 | Developmental coordination disorder |
| 128810 | Developmental coordination disorder |
| 128811 | Developmental coordination disorder |
| 128812 | Developmental coordination disorder |
| 128813 | Developmental coordination disorder |
| 128814 | Developmental coordination disorder |
| 128815 | Developmental coordination disorder |
| 128816 | Developmental coordination disorder |
| 128817 | Developmental coordination disorder |
| 128818 | Developmental coordination disorder |
| 128819 | Developmental coordination disorder |
| 128826 | Developmental coordination disorder |
| 128827 | Developmental coordination disorder |
| 128828 | Developmental coordination disorder |
| 128829 | Developmental coordination disorder |
| 128832 | Developmental coordination disorder |
| 128833 | Developmental coordination disorder |
| 128837 | Developmental coordination disorder |
| 129032 | Other cerebellar ataxia |
| 131005 | Ataxias, sensory |
| 131159 | Ataxia, sensory |
| 131163 | Ataxias, motor |
| 131711 | Ataxia, unspecified |
| 131712 | Lack of coordination |
| 131738 | Lack of coordination |
| 131739 | Lack of coordination |
| 131740 | Lack of coordination |
| 131742 | Lack of coordination |
| 131743 | Lack of coordination |
| 131744 | Lack of coordination |
| 131745 | Lack of coordination |
| 131746 | Lack of coordination |
| 131747 | Lack of coordination |
| 131786 | Ataxia, unspecified |
| 131787 | Lack of coordination |
| 131790 | Other lack of coordination |
| 131791 | Lack of coordination |
| 131805 | Other lack of coordination |
| 131806 | Lack of coordination |
| 131809 | Lack of coordination |
| 131810 | Lack of coordination |
| 149431 | Other late effects of cerebrovascular disease, ataxia |
| 182662 | Lack of coordination |
| 182663 | Lack of coordination |
| 182664 | Ataxia, unspecified |
| 191869 | Ataxia muscularis |
| 192711 | Spinocerebellar disease, unspecified |
| 193151 | Other late effects of cerebrovascular disease, ataxia |
| 193152 | Lack of coordination |
| 197060 | Other late effects of cerebrovascular disease, ataxia |
| 197061 | Lack of coordination |
| 251025 | Other cerebellar ataxia |
| 296610 | History of cerebellar ataxia |
| 382261 | Ataxia, unspecified |
| 383328 | Ataxia following nontraumatic intracerebral hemorrhage |
| 411202 | Ataxia, unspecified |
| 411203 | Static ataxia |
| 411211 | Ataxia, unspecified |
| 411212 | Ataxia, unspecified |
| 411213 | Lack of coordination |
| 414894 | Ataxia, unspecified |
| 426735 | Intermittent ataxia |
| 426948 | Ataxia, unspecified |
| 470958 | Ataxia, unspecified |
| 480502 | Ataxia, unspecified |
| 487866 | Ataxia, unspecified |
| 535450 | Cerebellar ataxia in diseases classified elsewhere |
| 539073 | Late-onset cerebellar ataxia |
| 539331 | Ataxia, unspecified |
| 1137633 | Ataxia, unspecified |
| 1319160 | Other cerebellar ataxia |
| 1430447 | Late-onset cerebellar ataxia |
| 1442192 | Lack of coordination |
| G83.89 | Cerebral paresis with homolateral ataxia |
| V12.40 | History of cerebellar ataxia |
| 333.79 | Symptomatic torsion dystonia |
| 3449 | Spasmodic torticollis |
| 60388 | Torticollis, spasmodic |
| 177607 | Cervical dystonia |
| 183478 | Isolated cervical dystonia |
| 534266 | Spasmodic torticollis |
| 1259437 | Spasmodic torticollis as late effect of trauma |
| G24.3 | Spasmodic torticollis |
| 44798 | Choreas |
| 44799 | Choreic movement |
| 44800 | Choreiform movement |
| 93979 | Chorea |
| 118502 | Choreic movements |
| 118504 | Movements, choreic |
| 118506 | Movements, choreiform |
| 118508 | Movement, choreic |
| 118510 | Movement, choreiform |
| 131671 | CHOREIFORM MOVEMENTS |
| 44939 | CLONUS |
| 333.81 | Blepharospasm |
| 333.82 | Meige syndrome (blepharospasm with oromandibular dystonia) |
| 3447 | Blepharospasm |
| 96736 | Blepharospasm syndrome |
| 159973 | Orofacial dystonia |
| 307638 | Idiopathic orofacial dystonia |
| 378020 | Meige syndrome (blepharospasm with oromandibular dystonia) |
| 378054 | Meige's syndrome (blepharospasm with oromandibular dystonia) |
| 432309 | Oromandibular dystonia |
| 502089 | Isolated oromandibular dystonia |
| 521315 | Benign essential blepharospasm |
| 534265 | Blepharospasm |
| 606675 | Idiopathic orofacial dystonia |
| 1426124 | Craniofacial dystonia |
| G24.4 | Idiopathic orofacial dystonia |
| G24.5 | Benign essential blepharospasm |
| IMO0002 | Craniofacial dystonia |
| 30572 | Neuroleptic-induced Parkinsonism |
| 30740 | Parkinsonism due to drug |
| 30742 | MPTP-induced Parkinsonism |
| 73160 | Parkinsonism due to drugs |
| 107249 | Drug-induced parkinsonism |
| 111458 | Methyl phenyl tetrahydropyridine induced parkinsonism |
| 111611 | MPTP (methyl phenyl tetrahydropyridine induced parkinsonism) |
| 123334 | Drug-induced Parkinson's disease |
| 537740 | Neuroleptic induced Parkinsonism |
| 608559 | Other drug induced secondary Parkinsonism |
| 609461 | Secondary parkinsonism due to other external agents |
| 641389 | Secondary parkinsonism due to other external agents |
| 1137394 | Neuroleptic induced Parkinsonism |
| 1192196 | Other drug-induced secondary parkinsonism |
| 1192197 | Other drug induced secondary Parkinsonism |
| 30570 | Medication-induced movement disorder |
| 30571 | Medication-induced postural tremor |
| 177596 | Drug induced tremor |
| 185097 | Lithium-induced tremor |
| 185099 | Metoclopramide-induced tremor |
| 185103 | Tricyclic antidepressant-induced tremor |
| 185105 | Valproic acid-induced tremor |
| 187431 | Valproate-induced tremor |
| 192992 | Drug-induced tremor |
| 200101 | Drug-induced tremor |
| 200102 | Drug induced tremor |
| 232438 | Metoclopramide-induced facial dystonia |
| 255770 | Tremor due to multiple drugs |
| 293367 | Drug-induced movement disorder |
| 539301 | Drug-induced tremor |
| 1181499 | Other drug induced movement disorders |
| 1185031 | Drug induced movement disorder, unspecified |
| 1350397 | Tremor due to substance abuse |
| G25.1 | Drug-induced tremor |
| E980.5 | Drug-induced tremor |
| 331.82 | LBD (Lewy body dementia) |
| 16500 | Dementia with Lewy bodies |
| 137071 | Lewy body dementia |
| 137072 | Lewy body disease |
| 168965 | Lewy body dementia with behavioral disturbance |
| 168967 | Lewy body dementia without behavioral disturbance |
| 251495 | Dementia, Lewy body with behavior disturbance |
| 327434 | LBD (Lewy body dementia) |
| 328861 | Autosomal dominant Lewy body dementia |
| 537979 | Dementia with Lewy bodies |
| 1233589 | Dementia with Lewy bodies (CODE) |
| G31.83 | Dementia with Lewy bodies |
| 238.4 | Dystonia Parkinsonism with hypermanganesemia, polycythemia, and chronic liver disease |
| 275.2 | Dystonia Parkinsonism with hypermanganesemia, polycythemia, and chronic liver disease |
| 333.6 | X-linked dystonia Parkinsonism |
| 571.8 | Dystonia Parkinsonism with hypermanganesemia, polycythemia, and chronic liver disease |
| 177617 | X-linked dystonia Parkinsonism |
| 515803 | Infantile Parkinsonism-dystonia |
| 533029 | Rapid onset dystonia parkinsonism |
| 533242 | Dystonia Parkinsonism with hypermanganesemia, polycythemia, and chronic liver disease |
| 533243 | Infantile dystonia Parkinsonism |
| 533255 | Infantile Parkinsonism with motor delay with tyrosine hydroxylase deficiency |
| 1349428 | Adult onset dystonia parkinsonism |
| 1350949 | Early onset dystonia parkinsonism |
| 1355108 | Hypermanganesemia with dystonia, polycythemia, and cirrhosis |
| D75.1 | Dystonia Parkinsonism with hypermanganesemia, polycythemia, and chronic liver disease |
| E83.41 | Dystonia Parkinsonism with hypermanganesemia, polycythemia, and chronic liver disease |
| G24.1 | X-linked dystonia Parkinsonism |
| K74.60 | Hypermanganesemia with dystonia, polycythemia, and cirrhosis |
| K76.89 | Dystonia Parkinsonism with hypermanganesemia, polycythemia, and chronic liver disease |
| 333.5 | Choreoathetosis with dystonia and pallidal atrophy |
| 333.8 | Fragments of torsion dystonia |
| 333.89 | Other fragments of torsion dystonia |
| 333.99 | Focal dystonia |
| 345.9 | Rolandic epilepsy with paroxysmal exercise induced dystonia and writer's cramp syndrome |
| 359.89 | Mitochondrial dystonia |
| 753.13 | Autosomal dominant DOPA-responsive dystonia |
| 758.5 | Myoclonic dystonia type 15 |
| 780.59 | Nocturnal paroxysmal dystonia |
| 799.89 | ANO3-related dystonia 24 |
| 3444 | Idiopathic torsion dystonia |
| 3446 | Fragments of torsion dystonia |
| 3451 | Other fragments of torsion dystonia |
| 30784 | Dystonia deformans progressiva |
| 30785 | Dystonia musculorum deformans |
| 30788 | Dystonia lenticularis |
| 46362 | Torsion dystonia |
| 61400 | Dystonia, torsion, fragments of |
| 61401 | Fragments of torsion dystonia |
| 98169 | Dystonia lenticularis syndrome |
| 102663 | Torsion dystonia fragment syndrome |
| 123857 | Dystonia |
| 135860 | Nocturnal paroxysmal dystonia |
| 135988 | Mitochondrial dystonia |
| 148746 | Dystonic movements |
| 153389 | Genetic torsion dystonia |
| 153392 | Symptomatic torsion dystonia |
| 153393 | Acquired torsion dystonia |
| 153394 | Torsion dystonia, acquired |
| 177614 | Dystonia due to DYT-1 gene mutation |
| 177615 | DOPA responsive dystonia |
| 177616 | DOPA-responsive dystonia |
| 177624 | Myoclonus dystonia |
| 177671 | Dystonia due to DYT1 gene mutation |
| 177672 | DRD (DOPA-responsive dystonia) |
| 177673 | Segawa's syndrome |
| 177879 | Focal dystonia |
| 177880 | Limb dystonia |
| 177881 | Segmental dystonia |
| 177882 | Hemidystonia |
| 193418 | Dystonia, unspecified |
| 197920 | Dystonia unspecified |
| 219767 | Torsion dystonia fragments |
| 225820 | Generalized dystonia |
| 251504 | DYT1 dystonia |
| 253045 | Dystonia of foot |
| 255468 | Task-specific dystonia of hand |
| 258050 | Paroxysmal dystonia |
| 308950 | Idiopathic nonfamilial dystonia |
| 315142 | Idiopathic familial dystonia |
| 383024 | Dystonic choreoathetosis |
| 397148 | Generalised dystonia |
| 419964 | Sleep-related dystonia |
| 425603 | Diurnal dystonia |
| 506145 | Dystonia 21 |
| 506612 | ANO3-related dystonia 24 |
| 510287 | Torsion dystonia type 21 |
| 515046 | Myoclonic dystonia type 15 |
| 515072 | Autosomal dominant torsion dystonia type 13 |
| 515185 | Dystonia type 12 |
| 515371 | Autosomal recessive torsion dystonia type 17 |
| 515398 | Torsion dystonia type 2 |
| 515481 | Adult onset focal torsion dystonia |
| 515490 | Dystonia musculorum deformans type 2 |
| 515510 | Torsion dystonia type 6 |
| 515584 | Torsion dystonia type 1 |
| 515656 | Dystonia type 23 |
| 515676 | Torsion dystonia type 4 |
| 515719 | Torsion dystonia type 7 |
| 515786 | Adult onset mixed type torsion dystonia |
| 515826 | X-linked torsion dystonia type 3 |
| 515876 | Dystonia musculorum deformans type 1 |
| 515890 | Dystonia type 9 |
| 523452 | Adult onset primary focal and segmental dystonia |
| 525657 | GTP cyclohydrolase 1 deficient DOPA-responsive dystonia |
| 525699 | Tyrosine hydroxylase-deficient DOPA-responsive dystonia |
| 525793 | DOPA-responsive dystonia with tyrosine hydroxylase deficiency |
| 526053 | DOPA responsive dystonia with autosomal dominant sepiapterin reductase deficiency |
| 527541 | Dystonia associated with mutation in ANO3 gene |
| 527542 | Dystonia associated with mutation in GNAL gene |
| 527543 | Dystonia associated with mutation in PRKRA gene |
| 527544 | PRKRA-related dystonia |
| 527663 | Dystonia type 25 |
| 528554 | Hereditary whispering dysphonia |
| 530585 | Rolandic epilepsy with paroxysmal exercise induced dystonia and writer's cramp syndrome (HCC) |
| 531034 | Choreoathetosis with dystonia and pallidal atrophy |
| 532720 | DOPA responsive dystonia with autosomal recessive sepiapterin reductase deficiency |
| 533043 | DOPA-responsive dystonia with guanosine triphosphate (GTP) cyclohydrolase 1 deficiency |
| 533044 | Dystonia with motor delay with guanosine triphosphate (GTP) cyclohydrolase 1 deficiency |
| 537830 | Dystonia |
| 538271 | Genetic torsion dystonia |
| 539524 | Dystonia, unspecified |
| 607601 | Idiopathic nonfamilial dystonia |
| 608560 | Other dystonia |
| 1137804 | Dystonia, unspecified |
| 1192198 | Other dystonia |
| 1261330 | Dystonia of extremity |
| 1274118 | Autosomal dominant DOPA-responsive dystonia |
| 1274181 | Primary dystonia DYT2 type |
| 1274208 | Autosomal recessive DOPA-responsive dystonia |
| 1274361 | Maternally inherited mitochondrial dystonia |
| 1274603 | Mitochondrial dystonia inherited from mother |
| 1350753 | Primary dystonia DYT4 type |
| 1350784 | Primary dystonia DYT13 type |
| 1350906 | Dystonia 16 |
| 1352855 | Infantile onset torsion dystonia |
| 1354454 | Juvenile onset dystonia |
| 1355562 | Generalized torsion dystonia |
| 1355813 | Autosomal dominant torsion dystonia type 4 |
| 1431080 | Hemidystonia and hemiatrophy syndrome |
| 1431170 | Hemidystonia-hemiatrophy syndrome |
| G24 | Dystonia |
| G24.2 | Idiopathic nonfamilial dystonia |
| G24.8 | Dystonia, unspecified |
| G24.9 | Dystonia, unspecified |
| G25.3 | Myoclonus dystonia |
| G25.5 | Choreoathetosis with dystonia and pallidal atrophy |
| G25.89 | Rolandic epilepsy with paroxysmal exercise induced dystonia and writer's cramp syndrome |
| G31.9 | Choreoathetosis with dystonia and pallidal atrophy |
| G40.909 | Rolandic epilepsy with paroxysmal exercise induced dystonia and writer's cramp syndrome |
| G47.8 | Nocturnal paroxysmal dystonia |
| G71.3 | Mitochondrial dystonia |
| G72.9 | Mitochondrial dystonia |
| Q61.2 | Autosomal dominant DOPA-responsive dystonia |
| R69 | Dystonia associated with mutation in ANO3 gene |
| V84.89 | Dystonia due to DYT-1 gene mutation |
| Z15.89 | Dystonia due to DYT-1 gene mutation |
| 21464 | Functional voice disorder |
| 72980 | Astasia-abasia, hysterical |
| 73051 | PSYCHOGENIC PARALYSIS |
| 78030 | Astasia-abasia |
| 104248 | Astasia-abasia syndrome |
| 113695 | Psychosomatic torticollis |
| 177642 | Psychogenic movement disorder |
| 177643 | Psychogenic gait |
| 252803 | Conversion disorder with motor symptoms or deficit |
| 387754 | Functional gait disorder with tremor |
| 428886 | Conversion disorder with abnormal movement |
| 428896 | Conversion disorder with speech symptoms |
| 428897 | Functional neurological symptom disorder with speech symptoms |
| 428898 | Conversion disorder with swallowing symptoms |
| 428899 | Functional neurological symptom disorder with swallowing symptoms |
| 428900 | Conversion disorder with weakness or paralysis |
| 428901 | Functional neurological symptom disorder with weakness or paralysis |
| 485797 | Conversion disorder with abnormal movement, acute episode, with psychological stressor |
| 485799 | Conversion disorder with abnormal movement, persistent, without psychological stressor |
| 485811 | Conversion disorder with weakness or paralysis, acute episode, with psychological stressor |
| 486482 | Conversion disorder with speech symptoms, acute episode, with psychological stressor |
| 486483 | Conversion disorder with speech symptoms, persistent, with psychological stressor |
| 486694 | Conversion disorder with weakness or paralysis, acute episode, without psychological stressor |
| 486909 | Conversion disorder with weakness or paralysis, persistent, with psychological stressor |
| 487055 | Conversion disorder with swallowing symptoms, persistent, with psychological stressor |
| 487142 | Conversion disorder with abnormal movement, acute episode, without psychological stressor |
| 487193 | Conversion disorder with weakness or paralysis, persistent, without psychological stressor |
| 487225 | Persistent mild somatic symptom disorder with predominant pain |
| 488205 | Somatic symptom disorder, persistent, mild, with predominant pain |
| 488240 | Somatic symptom disorder, persistent, moderate, with predominant pain |
| 488387 | Moderate somatic symptom disorder |
| 488388 | Somatic symptom disorder, moderate |
| 488393 | Mild somatic symptom disorder |
| 488394 | Somatic symptom disorder, mild |
| 488400 | Severe somatic symptom disorder |
| 488401 | Somatic symptom disorder, severe |
| 488406 | Persistent moderate somatic symptom disorder |
| 488407 | Somatic symptom disorder, persistent, moderate |
| 488411 | Persistent severe somatic symptom disorder |
| 488412 | Somatic symptom disorder, persistent, severe |
| 488427 | Persistent mild somatic symptom disorder |
| 488428 | Somatic symptom disorder, persistent, mild |
| 488512 | Conversion disorder, persistent episode, with swallowing symptoms, without psychological stressor |
| 488513 | Conversion disorder, persistent episode, with weakness or paralysis, with psychological stressor |
| 488530 | Conversion disorder, acute episode, with speech symptom, with psychological stressor |
| 488531 | Conversion disorder, acute episode, with speech symptom, without psychological stressor |
| 488533 | Conversion disorder, persistent episode, with speech symptom, with psychological stressor |
| 488534 | Conversion disorder, persistent episode, with speech symptom, without psychological stressor |
| 488548 | Conversion disorder, persistent episode, with abnormal movement, without psychological stressor |
| 488549 | Conversion disorder, persistent episode, with weakness or paralysis, without psychological stressor |
| 488561 | Conversion disorder, persistent episode, with abnormal movement, with psychological stressor |
| 488583 | Conversion disorder, persistent episode, with swallowing symptoms, with psychological stressor |
| 489015 | Conversion disorder with abnormal movement, acute episode |
| 489016 | Conversion disorder, acute episode, with abnormal movement |
| 489458 | Conversion disorder with speech symptoms, acute episode |
| 489459 | Conversion disorder, acute episode, with speech symptom |
| 489534 | Conversion disorder with weakness or paralysis, acute episode |
| 489535 | Conversion disorder, acute episode, with weakness or paralysis |
| 489578 | Conversion disorder with swallowing symptoms, acute episode |
| 489579 | Conversion disorder, acute episode, with swallowing symptoms |
| 1478697 | Functional neurological symptom disorder (conversion disorder), with abnormal movement |
| F44.4 | Conversion disorder with motor symptom or deficit |
| 306.0 | Musculoskeletal malfunction arising from mental factors |
| 177595 | Psychogenic tremor |
| 253297 | Functional tremor |
| 387754 | Functional gait disorder with tremor |
| 428887 | Functional neurological symptom disorder with abnormal movement |
| 1478697 | Functional neurological symptom disorder (conversion disorder), with abnormal movement |
| F45.8 | Other somatoform disorders |
| 333.4 | Huntington chorea |
| 49734 | Huntington chorea |
| 49735 | Huntington disease |
| 99039 | HC (Huntington chorea) |
| 99040 | HD (Huntington chorea) |
| 119882 | Akinetic-rigid variant of Huntington disease |
| 119883 | Huntington disease, akinetic-rigid variant |
| 119884 | Huntington disease, juvenile |
| 119885 | Huntington disease, juvenile-onset |
| 119886 | Huntington disease, late-onset |
| 119887 | Huntington disease, late onset |
| 119889 | Huntington disease, juvenile onset |
| 119890 | Juvenile Huntington disease |
| 119891 | Juvenile onset Huntington disease |
| 119892 | Juvenile-onset Huntington disease |
| 119895 | Late-onset Huntington disease |
| 119896 | Late onset Huntington disease |
| 411647 | Dementia due to Huntington chorea |
| 411983 | Dementia due to Huntington chorea |
| 527639 | Adult onset Huntington disease |
| 527640 | Adult onset Huntington chorea |
| F02.80 | Dementia due to Huntington chorea |
| G10 | Huntington disease |
| 331.5 | Idiopathic normal pressure hydrocephalus |
| 17423 | Idiopathic normal pressure hydrocephalus |
| 168971 | Idiopathic normal pressure hydrocephalus (INPH) |
| 168972 | Norml pressure hydroceph |
| 168973 | Hakim syndome |
| 168974 | Hakim syndrome |
| 168975 | Hakims syndrome |
| 168976 | Hakim's syndrome |
| 168977 | Hydrocephalus, normal pressure |
| 168978 | NPH (normal pressure hydrocephalus) |
| 168979 | Normal pressure hydrocephalus |
| 168980 | Normal pressure hydrocephalus (NPH) |
| 168981 | Normal pressure hydrocephalus syndrome |
| 168982 | Normal-pressure hydrocephalus |
| 168983 | Pressure hydrocephalus, normal |
| 168984 | Syndrome, Hakim |
| 168985 | Syndrome, Hakim's |
| 168986 | Normal pressure hydrocephalus NOS |
| 168987 | Hydrocephalus, low pressure |
| 168988 | Low pressure hydrocephalus |
| 169246 | Water on the brain |
| 191225 | INPH (idiopathic normal pressure hydrocephalus) |
| 218818 | Hydrocephalus, idiopathic normal pressure |
| 586834 | (Idiopathic) normal pressure hydrocephalus |
| 1176847 | (Idiopathic) normal pressure hydrocephalus |
| G91.2 | Idiopathic normal pressure hydrocephalus |
| 68900 | Tremor, physiological |
| 182707 | Physiological tremor |
| 328439 | Excessive physiologic tremor |
| 361220 | Anxiety related tremor |
| 361221 | Tremor, anxiety related |
| 333.83 | Spasmodic torticollis as late effect of trauma |
| 1114250 | Spasmodic torticollis due to infection |
| 331.3 | Secondary normal pressure hydrocephalus |
| 168969 | Secondary normal pressure hydrocephalus |
| G91.0 | Secondary normal pressure hydrocephalus |
| 3436 | Secondary parkinsonism |
| 30741 | Postencephalitic parkinsonism |
| 54085 | Paralysis agitans, secondary |
| 54087 | Parkinsonism, secondary |
| 112709 | Parkinson disease, postencephalitic |
| 113390 | Post-encephalitic Parkinson disease |
| 121254 | Von Economo encephalitis type Parkinsonism |
| 127243 | Postencephalitic Economo type Parkinsonism |
| 177645 | Psychogenic Parkinsonism |
| 299164 | Parkinsons disease, secondary |
| 539435 | Secondary parkinsonism, unspecified |
| 1137726 | Secondary parkinsonism, unspecified |
| 1192373 | Other secondary parkinsonism |
| 1216816 | Secondary parkinsonism, unspecified secondary Parkinsonism type |
| 1350604 | Parkinsonism after infection |
| 1350798 | Parkinsonism due to HIV infection |
| 1351512 | Parkinsonism concurrent with and due to acute infection |
| 1353200 | Charcot-Marie-Tooth disease with ptosis and parkinsonism |
| 1430171 | Infection as cause of parkinsonism |
| 1430667 | Parkinsonism due to mass lesion of brain |
| 1432145 | Parkinsonism due to and not concurrent with head injury |
| G21.9 | Secondary parkinsonism |
| 66566 | Cerebellar tremor |
| 66567 | Hunt's tremor |
| 131803 | Rubral tremor |
| 131804 | Rubral tremors |
| 163598 | Progressive cerebellar tremor |
| 177599 | Primary writing tremor |
| 177604 | Neuropathic tremor |
| 185101 | Caffeine-induced tremor |
| 1114378 | Tremor due to orthostatic hypotension |
| 1350397 | Tremor due to substance abuse |
| 1350475 | Tremor due to metabolic disorder |
| 1351412 | Tremor due to drug withdrawal |
| 68896 | Presenile tremor syndrome |
| 122358 | Senile tremor |
| 122367 | Senile tremors |
| 128916 | Secondary Parkinson disease |
| 128917 | Secondary vascular Parkinson disease |
| 145810 | Arteriosclerotic Parkinsonism |
| 158838 | Atherosclerotic Parkinsonism |
| 182803 | Vascular parkinsonism |
| G21.4 | Atherosclerotic Parkinsonism |
| 49425 | Insert peritoneal-venous shunt |
| 78291 | NM peritoneal venous shunt patency test |
| V45.2 | Ventriculo-peritoneal shunt status |
| Z98.2 | Ventriculo-peritoneal shunt status |
| 246687 | Atypical parkinsonism |
| 325436 | Parkinson's plus syndrome |
| 361246 | Dementia in Parkinson's plus syndrome |
| 1429790 | Atypical juvenile parkinsonism |
| 1484397 | Parkinsonism plus syndrome |
| 333430 | Parkinsonian syndrome associated with idiopathic orthostatic hypotension |
| 333431 | Parkinsonian syndrome associated with symptomatic orthostatic hypotension |
| 419686 | Parkinsonism with orthostatic hypotension |
| 1348911 | Orthostatic hypotension due to Parkinson's disease |
| 331.6 | Corticobasal degeneration |
| 334.2 | Corticobasal syndrome |
| 18936 | Corticobasal degeneration |
| 177605 | Corticobasal syndrome |
| 383090 | Dementia in corticobasal degeneration |
| 540115 | Progressive supranuclear ophthalmoplegia (steele-Richardson-olszewski) |
| 331.9 | Cerebral ventriculomegaly due to brain atrophy |
| 348.89 | Cerebral ventriculomegaly |
| 214626 | Cerebral ventriculomegaly |
| 259175 | Cerebral ventriculomegaly due to brain atrophy |
| G93.89 | Cerebral ventriculomegaly |
| 1264313 | Neurodegeneration with brain iron accumulation type 2A |
| Q99.8 | Neurodegeneration with brain iron accumulation type 2A |
| 30759 | Shy-Drager syndrome |
| 124425 | Shy-McGee-Drager syndrome |
| 161589 | Multiple system atrophy |
| 177589 | Multiple system atrophy-C |
| 177590 | Multiple system atrophy C |
| 177591 | Multiple system atrophy-P |
| 177592 | Multiple system atrophy P |
| 309553 | Multi-system degeneration of autonomic nervous system |
| 325435 | Parkinson's variant of multiple system atrophy |
| 327625 | Multiple system atrophy, Parkinson variant |
| 367392 | Multiple system degeneration of autonomic nervous system |
| 415682 | Multiple system atrophy with bradykinesia |
| 607964 | Multi-system degeneration of the autonomic nervous system |
| 1191974 | Multi-system degeneration of the autonomic nervous system |
| 1260226 | Multiple system atrophy with predominant parkinsonism |
| 1260291 | Multiple system atrophy with cerebellar features |
| G90.3 | Multi-system degeneration of autonomic nervous system |
| 333 | Olivocerebellar atrophy |
| 30705 | Olivocerebellar atrophy |
| 30748 | Olivopontocerebellar degeneration |
| 30750 | Olivopontocerebellar atrophy |
| 53344 | Olivo ponto cerebellar degeneration |
| 53345 | Olivo-ponto-cerebellar atrophy |
| 53346 | Olivo-ponto-cerebellar degeneration |
| 53347 | Olivo-ponto-cerebellar degenerations |
| 53348 | Olivopontocerebellar degenerations |
| 73161 | Olivopontocerebellar degeneration (Dejerine-Thomas syndrome) |
| 125965 | Familial olivopontocerebellar atrophy |
| 126302 | Idiopathic olivopontocerebellar atrophy |
| 126353 | Inherited olivopontocerebellar atrophy |
| 126971 | Nonfamilial olivopontocerebellar atrophy |
| 127206 | Pontoolivocerebellar atrophy |
| 148263 | OPCD (olivopontocerebellar degeneration) |
| 532706 | Olivopontocerebellar atrophy type 2 |
| 532772 | Olivopontocerebellar atrophy type 4 |
| 532773 | Olivopontocerebellar atrophy with dementia and extrapyramidal signs |
| 532774 | Olivopontocerebellar atrophy type 5 |
| 1274454 | Olivopontocerebellar degeneration with sensorineural hearing loss |
| 177598 | Orthostatic tremor |
| 30756 | Progressive supranuclear ophthalmoplegia |
| 30758 | Steele-Richardson-Olszewski syndrome |
| 31124 | Supranuclear paralysis |
| 95701 | Progressive supranuclear palsy |
| 112596 | Palsies, progressive supranuclear |
| 113565 | Progressive supranuclear palsies |
| 113680 | PSP (progressive supranuclear palsy) |
| 114934 | Supranuclear palsies, progressive |
| 114935 | Supranuclear palsy |
| 124381 | Richardson-Steele-Olszewski syndrome |
| 361287 | Dementia in progressive supranuclear ophthalmoplegia |
| 505991 | Autosomal dominant progressive supranuclear palsy |
| 538306 | Corticobasal degeneration |
| 1138075 | Progressive supranuclear ophthalmoplegia (steele-Richardson-olszewski) |
| 1353958 | Atypical progressive supranuclear palsy |
| G23.1 | Progressive supranuclear ophthalmoplegia |
| 332.1 | Parkinsonism due to hereditary spastic paraplegia |
| 515854 | X-linked Parkinsonism with spasticity |
| 1350673 | Parkinsonism due to hereditary spastic paraplegia |
| G11.4 | Parkinsonism due to hereditary spastic paraplegia |
| G21.8 | Parkinsonism due to hereditary spastic |
| ICD^*^ - International Classification of Diseases, EGD^**^ - Epic Diagnosis Groupers; DDX^***^ - Differential Diagnoses Generators | |
